# Birth and household exposures are associated with changes to skin bacterial communities during infancy

**DOI:** 10.1093/emph/eoae023

**Published:** 2024-09-17

**Authors:** Melissa B Manus, Maria Luisa Savo Sardaro, Omolola Dada, Maya Davis, Melissa R Romoff, Stephanie G Torello, Esther Ubadigbo, Rebecca C Wu, Maria Gloria Dominguez-Bello, Melissa K Melby, Emily S Miller, Katherine R Amato

**Affiliations:** Department of Anthropology, University of Texas at San Antonio, San Antonio, TX, USA; Department of Anthropology, Northwestern University, Evanston, IL, USA; Department of Anthropology, Northwestern University, Evanston, IL, USA; Department of Human Science and Promotion of the Quality of Life, University of San Raffaele, Rome, Italy; Department of Anthropology, Northwestern University, Evanston, IL, USA; Department of Anthropology, Northwestern University, Evanston, IL, USA; Department of Anthropology, Northwestern University, Evanston, IL, USA; Department of Anthropology, Northwestern University, Evanston, IL, USA; Department of Anthropology, Northwestern University, Evanston, IL, USA; Department of Anthropology, Northwestern University, Evanston, IL, USA; Department of Biochemistry and Microbiology, Rutgers University, New Brunswick, NJ, USA; Department of Anthropology, Rutgers University, New Brunswick, NJ, USA; Humans and the Microbiome Program, Canadian Institute for Advanced Research, Toronto, ON, Canada; Department of Anthropology, University of Delaware, Newark, DE, USA; Humans and the Microbiome Program, Canadian Institute for Advanced Research, Toronto, ON, Canada; Department of Obstetrics and Gynecology, Division of Maternal Fetal Medicine, Warren Alpert Medical School of Brown University, Providence, RI; USA; Department of Anthropology, Northwestern University, Evanston, IL, USA; Humans and the Microbiome Program, Canadian Institute for Advanced Research, Toronto, ON, Canada

**Keywords:** Skin microbiome, early life envrionments, antibiotics, mismatches

## Abstract

**Background and objectives:**

Microbial exposures during infancy shape the development of the microbiome, the collection of microbes living in and on the body, which in turn directs immune system training. Newborns acquire a substantial quantity of microbes during birth and throughout infancy via exposure to microbes in the physical and social environment. Alterations to early life microbial environments may give rise to mismatches, where environmental, cultural and behavioral changes that outpace the body’s adaptive responses can lead to adverse health outcomes, particularly those related to microbiome development and immune system regulation.

**Methods:**

This study explored the development of the skin microbiome among infants born in Chicago, USA. We collected skin swab microbiome samples from 22 mother-infant dyads during the first 48 h of life and again at 6 weeks postpartum. Mothers provided information about social environments and hygiene behaviors that may impact infants’ microbial exposures.

**Results:**

Analysis of amplicon bacterial gene sequencing data revealed correlations between infant skin bacterial abundances shortly after birth and factors such as antibiotic exposure and receiving a bath in the hospital. The composition of the infant microbiome at 6 weeks of age was associated with interactions with caregivers and infant feeding practices. We also found shifts in maternal skin microbiomes that may reflect increased hygiene practices in the hospital.

**Conclusions and implications:**

Our data suggest that factors related to the birth and household environment can impact the development of infant skin microbiomes and point to practices that may produce mismatches for the infant microbiome and immune system.

## BACKGROUND AND OBJECTIVES

Early life environments provide the infant body with exposures that are necessary for proper physiological development [1–4]. In particular, exposure to environmentally sourced microbes during infancy directly contributes to the development of the microbiome—the collection of microbes living in and on the body. The idea of a sterile womb has been challenged by evidence that supports microbial colonization beginning at birth [5–7] via microbial exposures from mothers’ birth canal, other body sites and the birth environment [8]. After birth, microbial colonization continues into infancy as physical and social environments expand [9, 10]. This period of microbial colonization is further influenced by stages of infant development, including breastfeeding, weaning and the introduction of solid foods [11, 12]. However, most early life microbiome research focuses on the effects of maternal-infant interactions on the microbial communities of the infant gut. As a result, there is a paucity of information on how additional aspects of early life environments impact the microbial communities of the organ that is in direct contact with the surrounding environment—the skin.

A central function of the microbiome during infancy is host immune system regulation. This includes promoting T-cell differentiation [13], influencing antibody and cytokine production [14, 15], and teaching the immune system to distinguish between commensal and pathogenic microbes [16]. Research in both human children and piglets shows that early life exposure to microbially rich outdoor settings (daycares and rearing facilities, respectively) contributes to skin microbial community composition and regulatory immune activity [17–19], just as work in mice shows that tolerance to skin commensals is achieved by microbial exposures during the first few weeks of life, but not after [14, 20]. These findings support the idea that microbial exposures during an early life ‘critical window’ can impact infant health in both the short and long term [3, 21].

As a corollary of the immune system’s reliance on early-life microbial exposures, changes to infants’ microbial environments may disrupt trajectories of immune system education. As initially described by the Hygiene Hypothesis [22] and expanded by the Old Friends Hypothesis [23, 24] and Disappearing Microbiota Hypothesis [25], environments that provide altered microbial exposures (i.e. exposures unlike those with which the human immune system evolved to interact) can yield poor immune regulation, with lasting effects on host health. This connection has been observed in human populations where lifestyle practices associated with urban living often restrict individuals’ microbial exposures [26–29]. For example, reduced contact with microbes in the natural environment is linked to allergy in urban-dwelling children [26, 29], while exposure to household pets (and their microbes) during infancy is correlated with a reduction in childhood atopy [27]. These connections suggest a *mismatch*—where rapid changes to microbial environments, often due to economic, industrial and cultural factors, outpace the body’s adaptive responses, which can lead to negative physiological and health outcomes [30, 31].

Many environmental mismatches that can alter infants’ microbial exposures are the unintended result of (largely beneficial) public health strategies. This includes the transition to indoor plumbing, heightened standards of personal hygiene and the widespread (mis)use of antibiotics. Though bathing and handwashing can disturb the communities of microbes on the skin [32], hygiene guidelines in many hospitals dictate that a newborn is bathed within the first 24 h of life to clean the skin and eliminate any pathogens acquired during delivery. Bathing (at delivery or later during infancy) may also alter the developing skin microbiome by physically removing microbes from the skin and/or altering skin pH, a factor to which microbes are sensitive. Similarly, many infants in the USA are administered prophylactic antibiotics, despite known associations between early life antibiotic exposure (and subsequent loss of commensal microbes) and the elevated risk of developing asthma and obesity in childhood [33, 34]. Antibiotics are also routinely administered during labor and delivery, even when their use is not indicated [35]. Taken together, these public health practices create birth and early life environments that are unlike the settings in which humans evolved and also contrast the practices of many contemporary non-industrialized populations where rates of asthma and obesity are lower than what is observed in the USA.

Additionally, the reductions in family and infant caregiving network sizes that are common in many industrialized settings may also create mismatched environments for infants. As cooperative breeders, a core component of the human reproductive and life history strategy is a reliance on assistance from non-maternal caregivers, or alloparents [36–38]. Given evidence that the skin microbiome is shaped by interactions with family and social group members across a range of host species [39–41], the highly social nature of human infancy suggests unique opportunities for human infants to acquire microbes from their alloparents [42–44], particularly during bouts of allocare (care provided by non-maternal caregivers) that involve direct skin-to-skin contact. It is currently unknown how these additional microbial exposures may complement those that stem from maternal care, or how changes to infants’ social environments may impact their health via alterations to the microbiome.

The COVID-19 pandemic catalyzed a confluence of behavioral changes that likely further contributed to environmental and microbial mismatches [45, 46]. For example, changes in social behaviors as a result of the pandemic (e.g. social distancing and isolation) may have curtailed the transmission of commensal microbes between individuals, including infants and their caregivers. For infants born to families who restricted their travel and attendance at family events, or adhered to local lockdowns, it is likely that their exposure to microbes from sources outside of the home, including the bodies of other individuals, was reduced. Additionally, increases in handwashing as well as sanitizing indoor surfaces could alter the quantity and/or types of microbes to which infants were exposed, in both hospital and household environments [47, 48]. However, there are limited data connecting these behaviors and practices to the infant microbiome in particular, which makes it difficult to predict how the pandemic affected microbial development and immune function during infancy.

This study leveraged questionnaire data and longitudinal skin swab samples from infants and mothers to address the question: how are early-life hygienic and social environments related to the development of bacterial communities across the infant skin? While this study included infants born during the COVID-19 pandemic, samples were collected after the pandemic began. As a result, we could not directly evaluate the effect of the pandemic on infant skin microbiomes, though we collected detailed data regarding hygiene practices and social interactions that could affect infants, regardless of when they are born. To address our research question, we first evaluated the hypothesis that the bacterial alpha diversity of infant skin shortly after birth varies based on antibiotic exposure, bathing practices, and body site (H1). We also hypothesized that skin bacterial alpha diversity during infancy is associated with infants’ social environments and bathing practices and differs by body site (H2). Finally, we expected that the bacterial alpha diversity and composition of infant skin varies over time (H3a), and to a greater extent than maternal skin (H3b). These hypotheses offer opportunities to explore evolutionarily novel behavioral and lifestyle practices that may create mismatches in the development of the microbiome during infancy.

## METHODOLOGY

Between April and June of 2021, participants (*N* = 22 mother–newborn dyads) were recruited on the postpartum floor of a hospital in Chicago, IL, USA. Electronic medical records of mothers and newborns were screened for participant eligibility. Exclusion criteria included cesarean delivery; current COVID-19 infection as indicated by a positive test at the hospital; permanent residence outside of Chicago or the immediate suburbs; the need for an English interpreter; a documented health condition (e.g. maternal diabetes or hypertension); or newborn admittance to the neonatal intensive care unit . Eligible mothers were contacted in-person in their hospital room by MBM and introduced to the study. The full study protocol was approved by the Institutional Review Board of Northwestern University (study number #STU00210184). Throughout this paper, we use the terms ‘mother’ and ‘maternal’ in relation to the participants in the current study but opt for gender-neutral terms when discussing broader implications. We use ‘bacteria’ and ‘bacterial’ to describe the results of the current study, which characterized the bacterial composition of the infant microbiome (but not the fungal or viral components), whereas ‘microbiome’, ‘microbe’ and ‘microbial’ refer to broader trends in the literature. We use the term ‘father’ to describe the biologically male co-parent of infants in this study.

### Sample collection

After informed consent was obtained from interested mothers on behalf of themselves and their newborns, MBM collected skin swab samples from mothers and newborns. The samples collected at the hospital are referred to as time point 1 (T1) throughout the paper. All T1 samples were collected on the day of delivery or the subsequent day. MBM contacted mothers via email to arrange a time for obtaining the follow-up samples at 6 weeks postpartum (T2). Due to logistical constraints, one family participated around 4 weeks postpartum. Follow-up samples were collected either at a park in Chicago or at participants’ homes (often outdoors). Five families were lost to attrition, while a sixth could not be reached for follow-up until 6 months postpartum. We excluded this sample from T2 analyses, which resulted in a sample size of 16 mother–infant dyads. The infant age range at T2 was 32–51 days, with an average age of 42.9 days. Details of sample collection are outlined in Table 1.

**Table 1.** Details of sample collection at T1 and T2

Timepoint	Infant age (days)	*N* mother-infant dyads	Location
T1	0–1	22	Hospital
T2	32–51 (mean = 42.9)	16	Participant’s home or public park

Regardless of sample location or timepoint, all skin swab samples were collected using the same protocol. A sterile dual-tipped cloth swab (Fisher BD BBL Media-free Sterile Swab) was vigorously rubbed on each body site for 1 min. Swabs were dipped into a solution of 0.15 M NaCl and 0.1% Tween 20 upon removal from the sterile plastic containers and immediately placed on the skin in order to minimize contamination from the sampling environment. Skin swab samples were collected from infants’ hand (palm), axilla (armpit) and outer cheek and mothers’ hand (palm), outer cheek and chest (across the clavicle). Swabs were returned to the plastic containers after sample collection, at which point the plastic container was stored in a cooler with ice. To confirm that there was no contamination from microbes in the surrounding air, control samples were taken from each sample collection location by swirling a swab in the air for 1 min. MBM followed COVID-19 safety protocols throughout the study, including collecting samples outdoors and wearing personal protective equipment  (face masks and gloves) during interactions with participants. All samples were immediately stored on ice for no more than 5 h before transport to the Amato Laboratory at Northwestern University, where they were stored in a −80˚ freezer until DNA extraction.

#### Q*uestionnaires*

At both timepoints, mothers completed a detailed questionnaire about their infants’ physical and social environments. MBM designed and administered the questionnaires using the interactive Network Canvas software [49]. At T1, the questionnaire focused on pregnancy and delivery, including mothers’ and newborns’ exposure to antibiotics. These questions included ‘Did you take any antibiotics during your pregnancy?’ and ‘Did you or your infant receive any antibiotics during or soon after labor?’ In the population from which our participants were drawn, antibiotics are administered during labor to pregnant people colonized with Group B *Streptococcus* [50] and to newborns with clinical suspicion of infection. Since newborns also routinely receive a bath in the first 24 h of life, we collected information on whether the infant had been bathed before skin swabbing.

At T2, the questionnaire addressed infants’ household and social environments. In addition to providing information about feeding practices and the presence of pets and siblings in the household, mothers generated a list of individuals who had ‘substantial social contact’ with their infant. Based on the list of names provided by each mother, the questionnaire then asked for demographic information of each caregiver, as well as the type of interactions that they have with the focal infant during a ‘typical day’. Individuals who were reported to perform at least one specific caregiving behavior (feeding; holding and carrying; playing; skin-to-skin contact; bathing and co-sleeping (defined as napping or sleeping overnight in direct contact with the infant)) were categorized as an alloparent. Since each infant in the study received consistent care from their father, we defined alloparent as a caregiver other than a genetic parent who was reported to perform at least one caregiving behavior on a typical day. Finally, we collected information on current antibiotic usage and the frequency and recency of infant bathing. A recent bath was defined as having occurred within 24 h of sample collection. Bath frequency (per week) was categorized into three groups for further analysis: less than once; 1–2 baths; or 3–6 baths.

#### Microbiome sample processing

DNA was extracted from the skin and air control samples using the Qiagen DNeasy PowerSoil Pro kit at the Amato Lab at Northwestern University. Extraction modifications for skin samples included warming the CD1 solution and modifying parameters during the vortex and centrifuge steps. The full extraction protocol can be found in Supplementary Appendix 1. The V3–V4 region of the 16S rRNA gene was amplified using a modified version of the Earth Microbiome Project protocol [51] and the 515 Fa/926R primer set [52, 53]. Amplicons were barcoded and pooled in equal concentrations for sequencing on the Illumina MiSeq V2 platform at the Genomics and Microbiome Core Facility at the Rush University Medical Center.

Paired-end sequences were joined and processed using QIIME2 v2020.6 [54] and sequences from mitochondria and chloroplasts were removed. 88 control samples (a combination of air controls and negative controls from DNA extraction and Polymerase Chain Reaction [PCR]) were included in the initial data set. After quality filtering and the removal of chloroplast and mitochondria sequences, the dada2 plug-in was used to cluster amplicon sequence variants (ASVs), and taxonomy was assigned by comparing ASVs to the GreenGenes13_8 reference database. This resulted in 9638 ASVs. Using previously published methods from our group [42], the taxonomic composition of the control samples and laboratory negatives were compared to the true (non-control) samples. There was no indication of contamination in the true samples by bacteria from the air, nor indication of contamination in the laboratory negatives. Based on these verifications, the air control and laboratory negative samples were removed from the dataset that was used in subsequent statistical analyses.

### Statistical analyses

The microbiome feature table was cleaned to remove any taxa assigned to the Order Chloroplast, the Family Mitochondria, or an ‘Unassigned’ Kingdom. This resulted in a dataset of 5 026 276 reads with an average of 22 439 reads/sample (range = 8506–322 160 reads/sample). Prior to analyses, the data were stratified by timepoint and then filtered to include only bacterial ASVs that were present in ≥ 10% of samples within each body site. Bacterial alpha and beta diversity (Shannon and Bray–Curtis, respectively) was estimated for non-rarefied data [55, 56] using the *phyloseq* package, version 1.44 [57] in R and version 4.2.2 [58]. Differences in alpha diversity were visualized using the *ggplot2* [59] and *ggsignif* [60] packages and tested using Wilcoxon rank sum tests (*stats* package). Pairwise permutational multivariate analysis of variance (PERMANOVA) models (between body sites within a given time point) were run using the *adonis* function in the *vegan* package, version 2.6.4 [61, 62] and bacterial community composition (beta diversity) was visualized with a non-metric multidimensional scaling (NMDS) plot using *ggplot2*. Redundancy analysis (RDA; with default parameters) in the *vegan* package [62] was used to explore associations between early life environmental variables and the centered log ratio (CLR)-transformed abundance profiles of bacterial ASVs on infant skin. The first RDA combined all infant skin sites at T1 and used the following predictors in univariable models (one model per predictor): body site, infant sex, maternal antibiotics at delivery, infant antibiotics at delivery and having had a bath before skin swabbing. The second RDA included the significant univariable predictors in a combined, multivariable model. We repeated this for each of the infant skin sites at T1, removing the ‘body site’ variable from the models. This approach was also applied to the infant skin samples at T2. The initial RDA included the same predictors as the model at T1 as well as: siblings, pets, bath frequency, bath recency, current pumping, current formula, current exclusive breastfeeding (direct consumption of human milk at the breast) and receiving allocare.

We then used PERMANOVA models to test if the predictors of bacterial abundance profiles in the RDA also had associations to skin beta diversity. This was repeated for all infant body sites combined, as well as within each body site. To avoid overfitting the T2 models, we ran one model using the T1 environmental variables as predictors (maternal antibiotics at delivery, infant antibiotics at delivery, and having had a bath prior to skin swabbing) and one model using the T2 environmental variables as predictors (infant sex, siblings, pets, bath frequency, bath recency, current pumping, current formula, current exclusive breastfeeding and receiving allocare). The *microViz* package, version 0.10.7 [63], was used to generate compositional heatmaps of CLR-transformed bacterial abundances (at the taxonomic level of family). These heatmaps were used to visualize the similarity, or clustering, of samples by body site within a given timepoint. Heatmaps were also used to display the Spearman correlations between CLR-transformed bacterial abundances and early life environmental variables. We defined moderate correlation between variables as a Spearman correlation coefficient between 0.30 and 0.60, while strong correlation was defined as a coefficient ≥ 0.60. At T1, the binary variables of interest included maternal antibiotics during labor, infant antibiotics shortly after delivery and having had a bath at the hospital prior to sample collection. At T2, the binary variables of interest included those analyzed at T1 as well as having at least one alloparent, having at least one sibling in the household, having at least one pet in the household, and bath recency (in the previous 24 h before sample collection). We also included a categorical variable related to per-week bathing frequency (less than once, 1–2 times, or 3–6 times).

Wilcoxon signed-rank tests were used to evaluate differences in average ASV prevalence (at the taxonomic level of genus) over time in both infant and maternal samples. Due to the compositional nature of microbiome data (i.e. errors are produced by having too many 0 s or 1 s), only the ASVs that were present in 12–88% of samples were included in each prevalence analysis. This filter was applied separately to maternal and infant samples. Differences in ASV prevalence were visualized with both plots and heat maps using ggplot2. UpSet plots were generated in the package *UpSetR* [64] and used to visualize bacterial taxonomic richness in infant and maternal samples at the two time points.

## RESULTS

This study included a total of 224 skin samples collected from 22 infant–mother dyads over two time points (perinatally and at ~6 weeks). Table 2 provides demographic information about the study participants. All infants lived with their mother and father, who provided regular care. In this study, every infant received regular care from their father, while five infants also received care from at least one alloparent other than the father. Of those five, two infants received allocare from an aunt, one infant received allocare from a grandmother and older brother (3 years of age), one infant received allocare from two older sisters (3 and 5 years of age) and one infant received allocare from two grandmothers and two grandfathers. Sibling alloparents lived in the same household as the focal infants, but adult alloparents (other than the father) did not. We were underpowered to analyze associations with maternal antibiotic usage during pregnancy (*N* = 1) as well as maternal (*N* = 2) and infant (*N* = 1) antibiotic use at T2. Though we were unable to control the timing of the first bath at the hospital, almost 1/3 of newborns were bathed prior to sample collection at T1 (Table 2).

**Table 2.** Participant demographics at (a) T1 and (b) T2; ratios and percentages are listed for binary variables (yes/no); maternal and infant age is shown with the range and average

T1	*N* newborns
Infant female sex	9/22 (41%)
Maternal age at delivery (years)	25-39 (33.3)
Maternal antibiotics during labor	7/22 (32%)
Neonatal antibiotics	4/22 (18%)
Hospital bath prior to sample collection	6/22 (27%)
Current exclusive breastfeeding^*^	8/19 (42%)

T2	*N* infants
Infant female sex	8/16 (50%)
Infant age (days)	32–51 (42.9)
Pet(s) in the household	10/16 (63%)
Sibling(s) in the household	6/16 (38%)
Receive allocare	5/16 (31%)
Any human milk feeding (direct breastfeeding or pumping)	11/14 (79%)
Frequent bath (3–6 per week)	5/16 (31%)
Recent bath (previous 24 ho)	4/16 (25%)
Current exclusive breastfeeding^*^	4/14 (29%)

^*^Feeding mode at time of sample collection; missing feeding data for 3 infants at T1and 2 infants at T2.

### Infant skin bacterial diversity and composition shortly after birth

**Figure 1.**
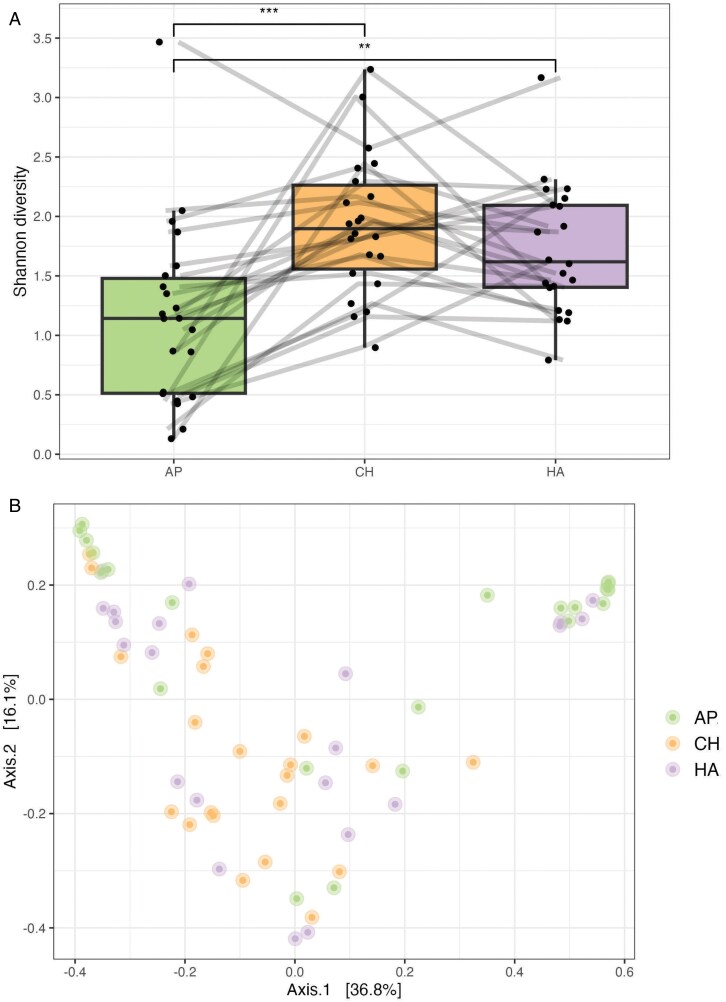
Infant (A) alpha and (B) beta diversity at T1. AP = axilla; CH = cheek and HA = hand; Wilcoxon rank sum test: ***P-value ≤ 0.001 and **P-value ≤ 0.01

The bacterial alpha diversity of infant samples at T1 varied by body site, with axilla samples displaying lower alpha diversity than the hand or cheek (Fig. 1a). Bacterial community composition did not vary significantly by infant skin site at T1 (Table 3; Fig. 1b). Univariable RDA models showed that body site and having had a bath prior to skin swabbing were significant predictors of bacterial abundance profiles (Table 4a) and that the body site variable explained the greatest amount of variation (9.3%). Body site and bath remained significant predictors in the multivariable model (Table 4b). In the univariable models of the infant axilla microbiome, none of the four variables (infant sex, maternal and infant antibiotic exposure and hospital bath prior to swabbing) significantly predicted bacterial abundance profiles (Table 4a). The bath variable was the lone significant univariable predictor of the cheek samples, while both the hospital bath and maternal antibiotics variables were significant univariable predictors of hand bacterial abundance profiles. The bath variable explained 12% and 2% of the variation in bacterial abundance profiles of the cheek and hand, respectively. In a multivariable model of the hand samples, the bath variable, but not maternal antibiotics, significantly predicted bacterial abundance profiles (Table 4b).

**Table 3.** Results of pairwise PERMANOVA of Bray–Curtis distances between infant skin sites at T1

Comparison	*R* ^2^* 100%	Pseudo-*F*	*P*-value
Axilla vs cheek	<1%	0.372	0.971
Axilla vs hand	<1%	0.297	0.989
Cheek vs hand	<1%	0.356	0.961

**Table 4.** (a) Univariable and (b) multivariable RDA results at T1; AP = axilla; CH = cheek;and HA = hand; bold values indicate statistically significant *P*-values

Body site	Predictor	*R* ^2^* 100%	pseudo-*F* value	*P*-value
(a) All body sites combined	Body site	9.30%	3.228	**<0.001**
	Infant sex	2.10%	1.349	0.055
	Maternal antibiotics during labor	2.10%	1.28	0.133
	Neonatal antibiotics	1.70%	1.061	0.369
	Hospital bath prior to sample collection	3.50%	1.985	**<0.001**
Infant AP	Infant sex	2.10%	0.429	0.955
	Maternal antibiotics during labor	5.20%	1.051	0.38
	Neonatal antibiotics	2.00%	0.384	0.327
	Hospital bath prior to sample collection	2.20%	0.377	0.795
Infant CH	Infant sex	2.70%	0.553	0.785
	Maternal antibiotics during labor	4.70%	0.927	0.426
	Neonatal antibiotics	4.10%	0.814	0.521
	Hospital bath prior to sample collection	12.00%	2.324	**<0.05**
Infant HA	Infant sex	1.20%	0.248	0.898
	Maternal antibiotics during labor	14.00%	3.104	**<0.05**
	Neonatal antibiotics	8.30%	1.723	0.147
	Hospital bath prior to sample collection	2.00%	4.372	**<0.01**
(b) All body sites combined	Combined model	8.00%	2.623	**<0.001**
	Body site	–	2.881	**<0.001**
	Hospital bath prior to sample collection	–	2.121	**<0.001**
Infant HA	Combined model	21.60%	3.348	**<0.05**
	Maternal antibiotics during labor	–	2.216	0.091
	Hospital bath prior to sample collection	–	4.48	**<0.01**

In a PERMANOVA of bacterial community composition (all infant skin sites combined), neither body site nor the bath variable were significant predictors, despite being significant predictors in the RDA of bacterial abundance profiles (Table 5). In contrast, both maternal and infant antibiotics at delivery showed significant associations with bacterial community composition (Table 5). These results did not persist when PERMANOVAs where stratified by each individual body site; in the stratified models, none of the four predictors had significant associations to the bacterial community composition of the infant axilla, hand, or cheek.

**Table 5.** Results of PERMANOVA of Bray-Curtis distances of infant skin sites at T1 using predictors from the RDA; AP = axilla; CH = cheek and HA = hand; bold values indicate statistically significant *P*-values

Body site	Predictor	*R* ^2^* 100%	Pseudo-*F*	*P*-value
All body sites combined	Body site	1.10%	0.293	0.999
	Infant sex	3.20%	1.72	0.104
	Maternal antibiotics during labor	6.00%	3.213	**<0.01**
	Neonatal antibiotics	6.00%	3.257	**<0.01**
	Hospital bath prior to sample collection	1.90%	1.039	0.349
Infant AP	Infant sex	4.60%	0.697	0.634
	Maternal antibiotics during labor	7.90%	1.211	0.241
	Neonatal antibiotics	3.10%	0.472	0.241
	Hospital bath prior to sample collection	5.00%	0.771	0.582
Infant CH	Infant sex	8.00%	1.388	0.175
	Maternal antibiotics during labor	5.40%	0.929	0.513
	Neonatal antibiotics	7.10%	1.222	0.279
	Hospital bath prior to sample collection	9.60%	1.658	0.081
Infant HA	Infant sex	13.20%	2.421	0.067
	Maternal antibiotics during labor	5.00%	0.919	0.462
	Neonatal antibiotics	6.50%	1.198	0.322
	Hospital bath prior to sample collection	5.70%	1.035	0.382

**Figure 2.**
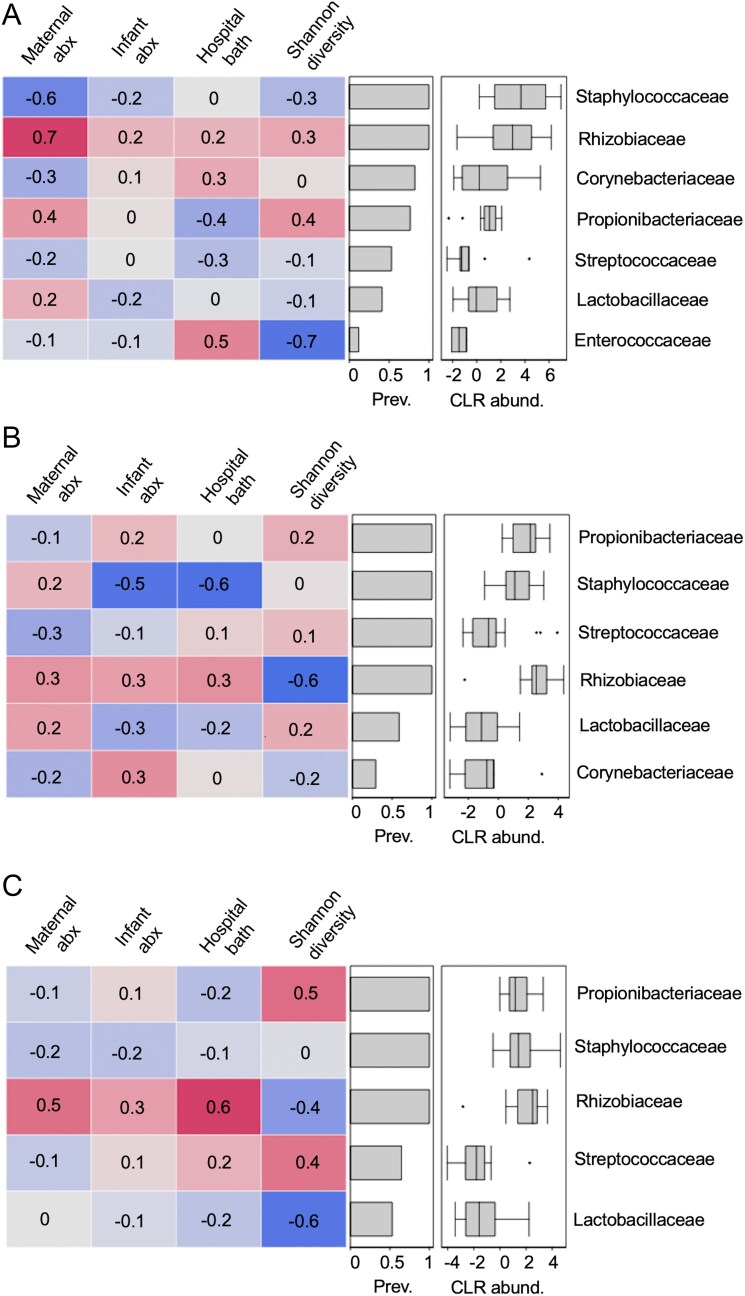
Spearman correlations between bacterial alpha diversity, early-life environmental variables, and the abundances of bacterial families at T1 in infant (A) axilla, (B) cheek and (C) hand samples; plots display bacterial families present in at least 5% of samples and at a summed read count of 8000 across samples (within each body site); prev. = prevalence and CLR_abund = CLR-transformed abundances

Infant antibiotic exposure at delivery was weakly correlated to the abundances of bacterial families on the infant axilla (Fig. 2), yet moderately correlated with *Staphylococcaceae* abundance on the infant cheek (Spearman rho = −0.50) and *Rhizobiaceae* abundance on the infant hand (Spearman rho = 0.30). Maternal antibiotic exposure during labor was strongly correlated to the abundances of *Staphylococcaceae* (Spearman rho = −0.60) and *Rhizobiaceae* (Spearman rho = 0.70) on the infant axilla (Fig. 2). This variable was moderately correlated to *Rhizobiaceae* abundance on the infant hand (Spearman rho = 0.50). Similarly, having had a bath in the hospital prior to skin swabbing had a moderate correlation to *Enterococcaceae* abundance (Spearman rho = 0.50) on the infant axilla, *Staphylococcaceae* abundance (Spearman rho = −0.60) on the infant cheek and *Rhizobiaceae* abundance (Spearman rho = 0.60) on the infant hand (Fig. 2).

### Infant skin bacterial alpha diversity and composition at 6 weeks of age

**Figure 3.**
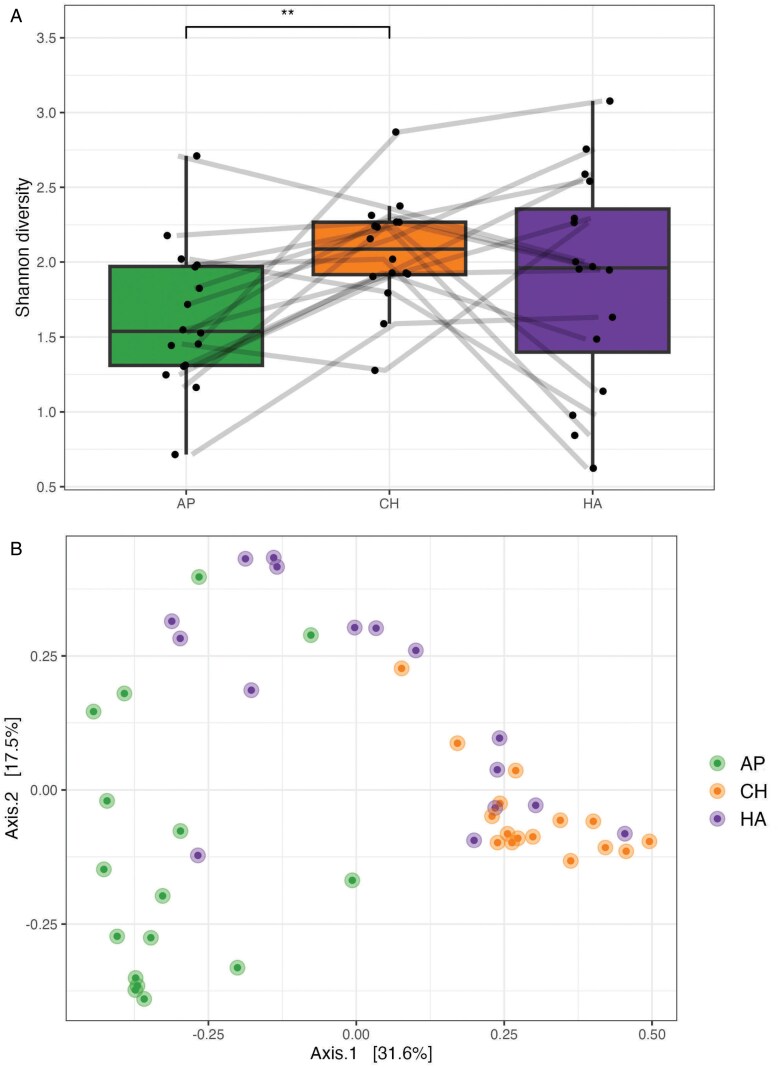
Infant (A) alpha and (B) beta diversity at T2. AP = axilla; CH = cheek and HA = hand; Wilcoxon rank sum test; **P-value ≤ 0.01

At T2, the bacterial alpha diversity of the infant axilla was significantly lower than that of the cheek and trended toward lower than the hand (Fig. 3a). The bacterial community composition of the infant cheek was significantly different from the other two body sites (Table 6; Fig. 3b). A univariable RDA model (all samples combined) showed that body site, infant sex and current formula feeding were significant predictors of bacterial abundance profiles (Table 7a) and that the body site variable explained the greatest amount of variation in abundance profiles at this timepoint (22.5%). None of the environmental variables from T1 were significant univariable predictors at T2. Body site and current formula feeding remained significant predictors in the multivariable model (Table 7b). None of the predictors showed a statistically significant relationship to bacterial abundance profiles when models were stratified by body site (Table 7a). There was a trending relationship between receiving allocare and the bacterial abundance profile of the infant axilla and hand (axilla: *R*^2^ = 13.4%; pseudo-*F* = 2.168; *P*-value = 0.089; hand: *R*^2^ = 14.5%; pseudo-*F* = 2.377; *P*-value = 0.077), as well as exclusive breastfeeding and the bacterial abundance profile of the infant hand (*R*^2^ = 15.5%; pseudo-*F* = 2.206; *P*-value = 0.083).

**Table 6.** Results of pairwise PERMANOVA of Bray–Curtis distances between infant skin sites at T2; bold values indicate statistically significant *P*-values

Comparison	*R* ^2^* 100%	Pseudo-*F*	*P*-value
Axilla vs cheek	7.80%	2.533	**<0.05**
Axilla vs hand	2.20%	0.684	0.689
Cheek vs hand	10.30%	3.449	**<0.01**

**Table 7.** (a) Univariable and (b) multivariable redundancy analysis (RDA) results at T2. EBF = exclusive breastfeeding; AP = axilla; CH = cheek and HA = hand; bold values indicate statistically significant *P*-values

Body site	Predictor	*R* ^2^* 100%	pseudo-*F* value	*P*-value
(a) All body sites combined	Body site	22.50%	6.52	**<0.001**
	Infant sex	3.70%	1.788	**<0.05**
	Maternal antibiotics during labor	2.70%	1.257	0.161
	Neonatal antibiotics	1.80%	0.766	0.766
	Hospital bath prior to sample collection	4.50%	0.989	0.46
	Sibling(s) in the household	2.30%	1.103	0.258
	Pet(s) in the household	10.50%	1.256	0.105
	Bath frequency	8.60%	1.018	0.435
	Bath recency	2.20%	0.971	0.432
	Current pumping	2.40%	1.003	0.382
	Current formula	4.00%	1.659	**<0.05**
	Current EBF	3.30%	1.136	0.117
	Receive allocare	2.70%	1.276	0.145
Infant AP	Infant sex	6.41%	0.959	0.423
	Maternal antibiotics during labor	2.52%	0.361	0.847
	Neonatal antibiotics	13.19%	1.958	0.096
	Hospital bath prior to sample collection	8.32%	0.545	0.8
	Sibling(s) in the household	8.73%	1.338	0.243
	Pet(s) in the household	23.84%	0.861	0.573
	Bath frequency	17.42%	0.58	0.8
	Bath recency	9.15%	1.309	0.348
	Current pumping	3.49%	0.435	0.802
	Current formula	1.51%	0.184	0.944
	Current EBF	9.13%	1.191	0.305
	Receive allocare	13.41%	2.168	0.089
Infant CH	Infant sex	9.38%	1.45	0.135
	Maternal antibiotics during labor	11.00%	1.729	0.274
	Neonatal antibiotics	3.02%	0.404	0.588
	Hospital bath prior to sample collection	4.47%	0.281	0.825
	Sibling(s) in the household	5.93%	0.883	0.485
	Pet(s) in the household	45.50%	2.296	0.166
	Bath frequency	7.01%	0.207	0.911
	Bath recency	19.50%	3.041	0.108
	Current pumping	9.31%	1.231	0.238
	Current formula	3.55%	0.441	0.375
	Current EBF	19.15%	2.843	0.11
	Receive allocare	12.80%	2.055	0.216
Infant HA	Infant sex	7.10%	1.062	0.321
	Maternal antibiotics during labor	10.82%	1.699	0.15
	Neonatal antibiotics	1.60%	0.206	0.965
	Hospital bath prior to sample collection	20.50%	1.554	0.198
	Sibling(s) in the household	6.40%	0.956	0.371
	Pet(s) in the household	24.50%	0.891	0.574
	Bath frequency	26.60%	0.995	0.5
	Bath recency	5.70%	0.8	0.472
	Current pumping	14.10%	1.971	0.114
	Current formula	2.80%	0.351	0.886
	Current EBF	15.50%	2.206	0.083
	Receive allocare	14.50%	2.377	0.077
(b) All body sites combined	Combined model	22.60%	3.989	**<0.001**
	Body site	–	5.974	**<0.001**
	Infant sex	–	1.901	**<0.05**
	Current formula	–	2.109	**<0.05**

Body site was the only significant predictor of bacterial community composition at T2 when all infant skin sites were analyzed together (Table 8). In the models stratified by body site, exclusive breastfeeding and having siblings in the household were significant predictors of bacterial community composition in infant cheek samples (Table 8). Receiving allocare and current pumping trended toward a relationship with bacterial community composition of the infant cheek (allocare: *R*^2^ = 13.4%, pseudo-*F* = 3.298; *P*-value = 0.057; pumping: *R*^2^ = 12.8%, pseudo-*F* = 3.148; *P*-value = 0.064).

**Table 8.** PERMANOVA of Bray–Curtis distances of infant skin sites at T2 using predictors from the RDA; for each of the body sites, the model was stratified into Model A and Model B to avoid overfitting, resulting in a total of eight models; EBF = exclusive breastfeeding; AP = axilla; CH = cheek and HA = hand; bold values indicate statistically significant *P*-values

Body site	Predictor	*R* ^2^* 100%	Pseudo-*F*	*P*-value
All body sites combined	Model A			
	Body site	8.95%	1.967	**<0.05**
	Maternal antibiotics during labor	2.98%	1.0594	0.385
	Neonatal antibiotics	15.80%	0.552	0.891
	Hospital bath prior to sample collection	68.80%	1.21	0.21
	Model B			
	Body site	11.19%	2.437	**<0.01**
	Infant sex	3.69%	1.6081	0.117
	Sibling(s) in the household	1.48%	0.6455	0.755
	Pet(s) in the household	4.51%	0.6538	0.905
	Bath frequency	2.92%	0.6358	0.86
	Bath recency	1.79%	0.7787	0.601
	Current pumping	1.63%	0.7105	0.679
	Current formula	1.76%	0.7647	0.638
	Current EBF	1.41%	0.6139	0.789
	Receive allocare	1.49%	0.6493	0.744
Infant AP	Model A			
	Maternal antibiotics during labor	4.83%	0.548	0.792
	Neonatal antibiotics	3.14%	0.3559	0.903
	Hospital bath prior to sample collection	9.72%	0.5511	0.861
	Model B			
	Infant sex	1.63%	0.139	0.984
	Sibling(s) in the household	6.68%	0.5664	0.741
	Pet(s) in the household	11.60%	0.3278	0.975
	Bath frequency	18.20%	0.7715	0.673
	Bath recency	6.57%	0.5576	0.77
	Current pumping	2.07%	0.1751	0.985
	Current formula	1.55%	0.1312	0.985
	Current EBF	2.32%	0.1967	0.978
	Receive allocare	6.54%	0.5544	0.763
Infant CH	Model A			
	Maternal antibiotics during labor	4.92%	0.6381	0.796
	Neonatal antibiotics	9.70%	1.2571	0.27
	Hospital bath prior to sample collection	18.47%	1.1965	0.269
	Model B			
	Infant sex	11.86%	2.9125	0.083
	Sibling(s) in the household	15.60%	3.8298	**<0.05**
	Pet(s) in the household	22.86%	1.871	0.189
	Bath frequency	22.54%	2.7662	0.079
	Bath recency	5.72%	1.4047	0.316
	Current pumping	12.82%	3.1481	0.064
	Current formula	8.65%	2.1226	0.171
	Current EBF	15.47%	3.7983	**<0.05**
	Receive allocare	13.43%	3.298	0.057
Infant HA	Model A			
	Maternal antibiotics during labor	5.65%	0.7219	0.66
	Neonatal antibiotics	5.31%	0.6781	0.712
	Hospital bath prior to sample collection	18.36%	1.1727	0.322
	Model B			
	Infant sex	7.97%	0.6426	0.723
	Sibling(s) in the household	2.25%	0.1811	0.988
	Pet(s) in the household	18%	0.4834	0.899
	Bath frequency	13.46%	0.5423	0.848
	Bath recency	4.84%	0.3899	0.89
	Current pumping	3.25%	0.2617	0.966
	Current formula	5.29%	0.4266	0.868
	Current EBF	3.36%	0.271	0.97
	Receive allocare	3.63%	0.2926	0.948

**Figure 4.**
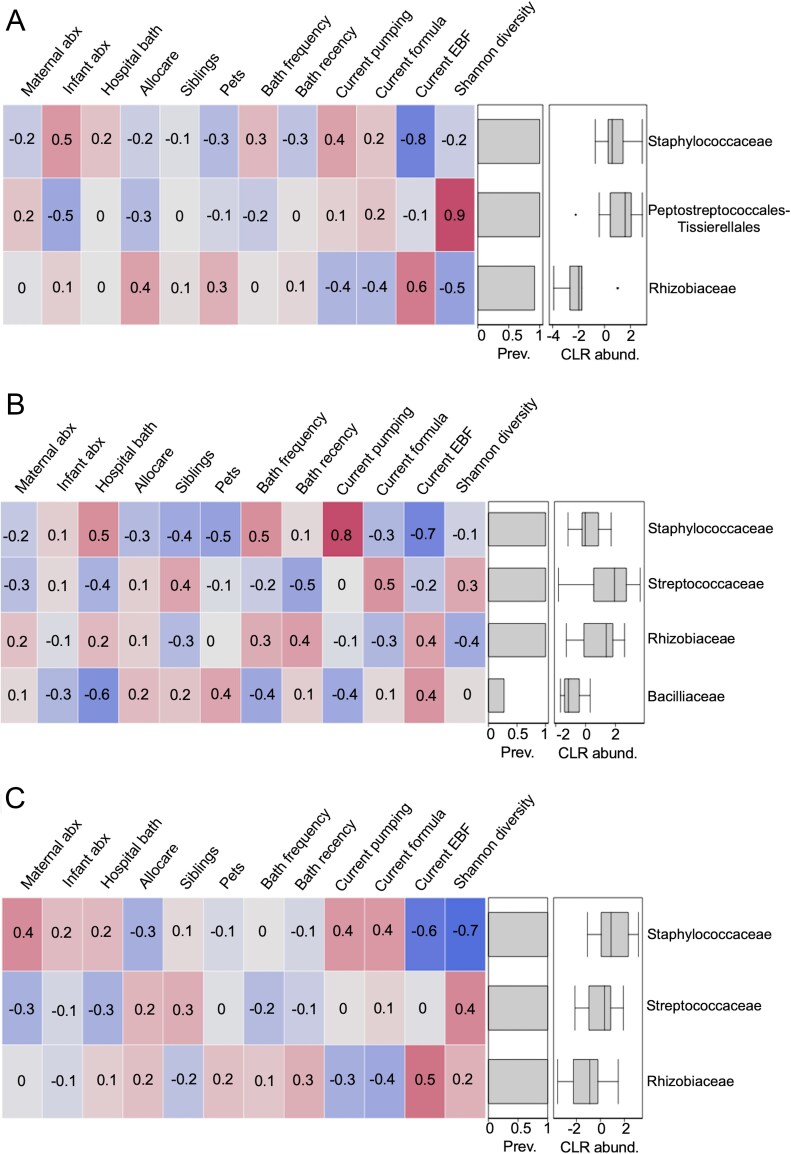
Spearman correlations between bacterial alpha diversity, early-life environmental variables, and the abundances of bacterial families at T2 in infant (A) axilla, (B) cheek and (C) hand samples; plots display bacterial families present in at least 5% of samples and at a summed read count of 8000 across samples (within each body site); rev. = prevalence and CLR_abund = CLR-transformed abundances

Bath recency was moderately negatively correlated to *Streptococcaceae* abundance (Spearman rho = −0.5) on the infant cheek at T2 (Fig. 4), while bath frequency was moderately positively correlated to the abundance of *Staphylococcaceae* (Spearman rho = 0.5). There were also moderate correlations between having a bath at the hospital prior to skin swabbing at T1 and the abundances of *Staphylococcaceae* (Spearman rho = 0.5) and *Bacillaceae* (Spearman rho = −0.6) on the infant cheek at T2 (Fig. 4). Newborn antibiotic exposure at birth was also moderately correlated to the abundances of *Staphylococcaceae* (Spearman rho = 0.5) and *Peptostreptococcales-Tissierellales* (Spearman rho = −0.5) on the infant axilla at T2. Additionally, exclusive breastfeeding was strongly negatively correlated to the abundances of *Staphylococcaceae* on the infant axilla (Spearman rho = −0.8), cheek (Spearman rho = −0.7) and hand (Spearman rho = −0.6). This variable was moderately correlated to *Rhizobiaceae* abundance on the infant axilla (Spearman rho = 0.6) and hand (Spearman rho = 0.5). *Staphylococcaceae* abundance on the infant cheek was also strongly correlated to receiving pumped milk at T2 (Spearman rho = 0.8). Receiving formula was moderately positively correlated to the abundance of *Streptococcaceae* (Spearman rho = 0.5) on the infant cheek.

### Variation in the infant and maternal skin microbiome during the first 6 weeks postpartum

**Figure 5.**
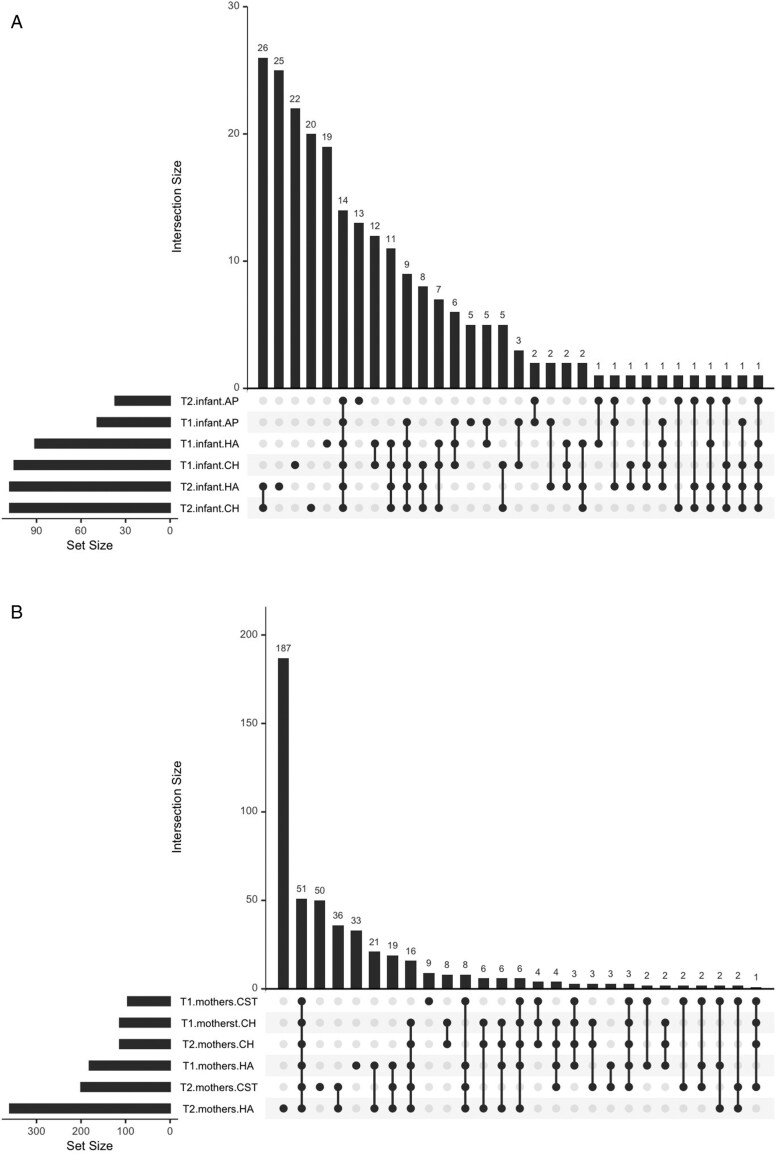
Distinct sets of bacterial ASVs shared across body sites and timepoints in (a) infant and (b) maternal samples. AP = axilla; CH = cheek; CST = chest and HA = hand; intersection size denotes the number of ASVs uniquely present in one or more groups of samples; ASVs present in only one sample group are displayed by a single dot, while uniquely shared sets of ASVs are denoted by vertical lines between dots; set size refers to the total number of ASVs in each sample group

**Figure 6.**
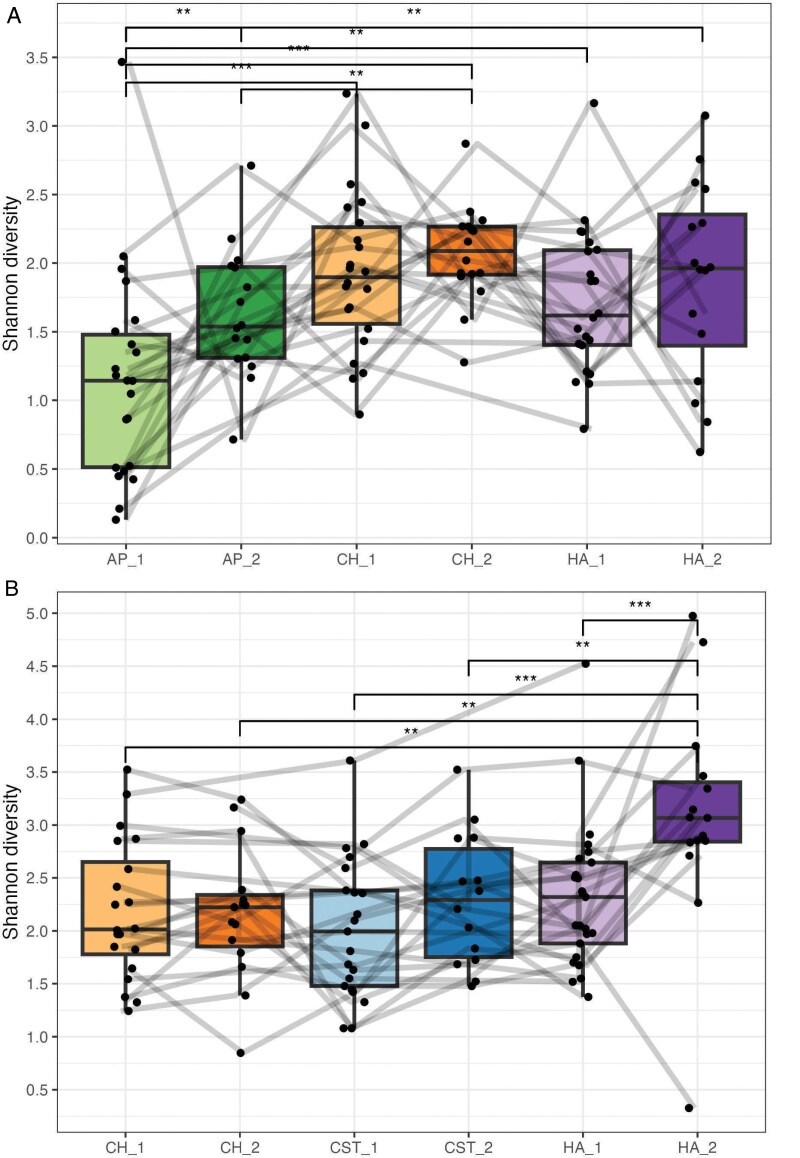
Bacterial alpha diversity of (A) infant and (B) maternal skin sites over time; lines connect samples collected from the same individual; AP = axilla; CH = cheek; CST = chest; HA = hand; 1 = T1 and 2 = T2; Wilcoxon rank sum test: ***P-value ≤ 0.001; **P-value ≤ 0.01 and *P-value ≤ 0.05

In both infant and maternal samples, bacterial richness differed by skin site and between the two time points (Fig. 5). At T1, the infant cheek harbored the largest unique set of bacterial ASVs (*N* = 22), followed by the hand (*N* = 19) and the axilla (*N* = 5) (Fig. 5a). The infant hand harbored 25 bacterial ASVs at T2, while the infant cheek and axilla displayed unique sets of 20 and 13 bacterial ASVs, respectively. Maternal hand samples harbored a larger unique set of bacterial ASVs (*N* = 33) compared to the chest (*N* = 9) at T1, while the cheek did not contain a unique set of bacterial ASVs not shared with other body sites (Fig. 5b). At T2, the difference in unique set sizes increased, with the maternal hand harboring 187 bacterial ASVs and the chest containing 50. Similar to T1, the maternal cheek did not display a unique set of bacterial ASVs at the second timepoint, though eight bacterial ASVs were uniquely shared between maternal cheek samples across T1 and T2. Bacterial alpha diversity displayed similar trends and varied by skin site (Fig. 6). For each skin site, there was a trend of increasing bacterial alpha diversity between the two time points, though only the differences in alpha diversity of the infant axilla and maternal hand reached statistical significance.

**Figure 7.**
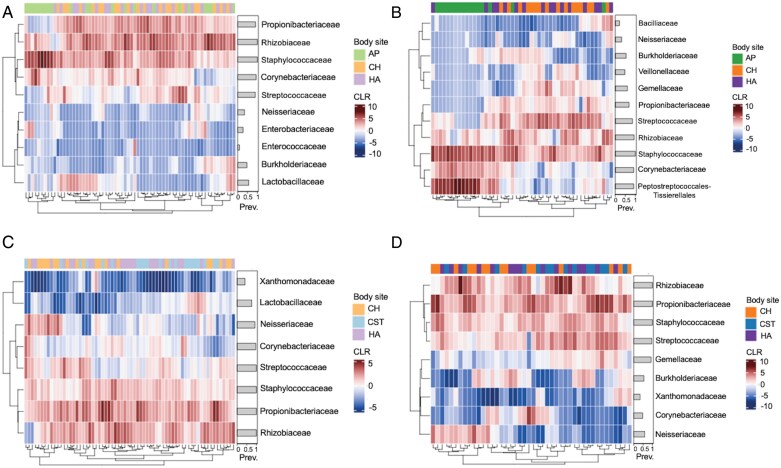
Hierarchical clustering by skin site of CLR-transformed bacterial abundances in (A) infant T1, (B) infant T2, (C) maternal T1 and (D) maternal T2 samples; plots include bacterial families present in at least 5% of all samples with a summed read count of 8000 (within each group a–d); AP = axilla; CH = cheek; CST = chest; HA = hand; Prev. = prevalence

Using bacterial abundance data, infant axilla samples clustered by composition at both time points. At T1, this was driven in part by a lower abundance of *Propionibacteriaceae* alongside a greater abundance of *Staphylococcaceae* compared to the hand and cheek samples (Fig. 7a). This trend became more pronounced at T2, where axilla samples had elevated abundances of *Peptostreptococcales-Tissierellales*, *Corynebacteriaceae* and *Staphylococcaceae*, yet lower abundances of *Streptococcaceae* and *Propionibacteriaceae*, compared to the other body sites (Fig. 7b). In contrast, the maternal samples did not cluster by body site as clearly as the infant samples (Fig. 7c and d). At T1, the majority of maternal samples showed an elevated abundance of *Staphylococcaceae*, *Propionibacteriaceae* and *Rhizobiaceae*, alongside a lower abundance of *Xanthomonadaceae* (Fig. 7c). This pattern was fairly consistent at the second timepoint (Fig. 7d). Some of the maternal samples displayed lower abundances of *Lactobacillus* at T1, yet this taxon was not dominant at T2 (based on prevalence thresholds used to subset bacterial taxa).

**Figure 8.**
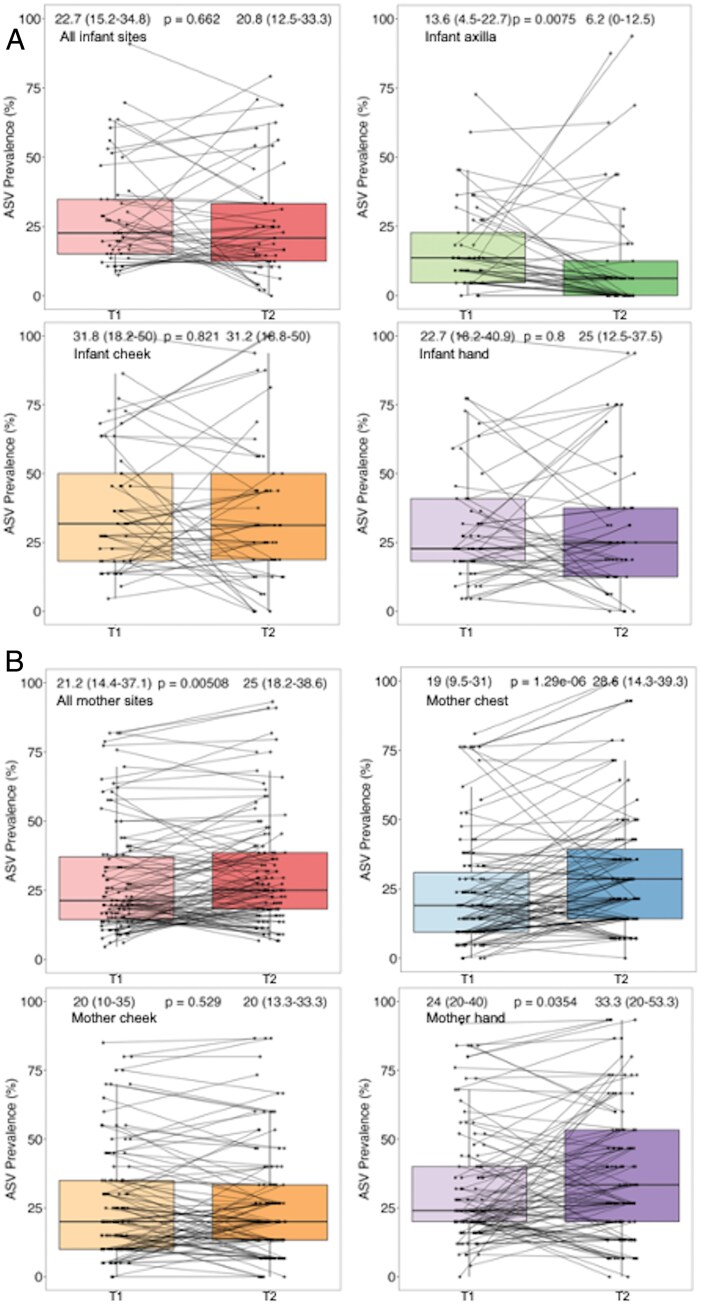

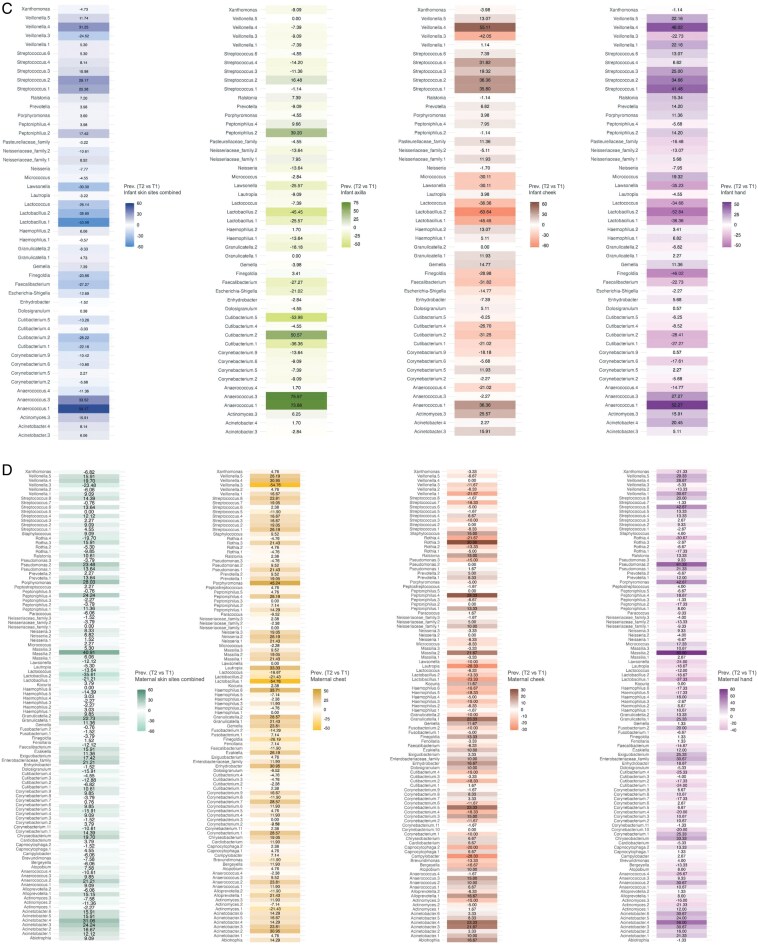
Difference in ASV prevalence (at the taxonomic level of Genus) between the two timepoints in infant and maternal samples; A and B: boxplots display the results of Wilcoxon tests of the difference in average ASV prevalence between T1 and T2; average prevalence, range, and *P*-value from the Wilcoxon test are shown; C and D: Heatmaps display the difference in prevalence between individual ASVs at T2 compared to T1; ASV were retained in the analysis if prevalent in 12–88% of samples stratified by infant or maternal origin; prev. = prevalence

Average bacterial ASV prevalence in infant samples (all skin sites combined) did not vary over time (Fig. 8), though individual ASVs displayed variation in prevalence between the two timepoints. For example, *Anaerococcus.1* was considerably more prevalent on infant skin at T2, while the prevalence of *Lactobacillus.2* decreased over time (Fig. 8c). When samples were stratified by infant body site, average ASV prevalence was significantly lower in the axilla samples collected at T2 (Fig. 8a). The largest increases in prevalence were found in *Anaerococcus.1*, *Anaerococcus.3* and *Cutibacterium.2*, while *Cutibacterium.5* and *Lactobacillus.2* displayed the largest decreases in prevalence over time. Average ASV prevalence did not vary over time in the cheek or hand samples (Fig. 8a), though certain ASVs did vary considerably between the two-time points (Fig. 8c). This included an increased prevalence of *Anaerococcus.1*, *Streptococcus.1* and *Veillonella.4*, alongside a decreased prevalence of *Lactobacillus.2* and *Finegoldia*, in hand samples, as well as variation in *Veillonella.4* (increased) and *Lactobacillus.2* (decreased) in cheek samples. Of note, *Veillonella.5* displayed a moderate increase in prevalence over time in the infant hand and cheek samples but was absent from all axilla samples.

In contrast, average ASV prevalence (all skin sites combined) was higher in maternal samples at T2 compared to T1 (Fig. 8b). This difference was driven by increased ASV prevalence in maternal chest and hand, but not cheek, samples. The individual ASVs (Fig. 8d) with the largest differences in prevalence between the two timepoints varied across maternal body sites. *Porphyromonas* (increased), *Lactobacillus.1* (decreased) and *Veillonella.3* (decreased) showed the largest variation in chest samples, while *Massilia.2* (increased), *Pseudomonas.2* (increased), *Acinetobacter.4* (increased) and *Lactobacillus.1* (decreased) were the most variable in hand samples. In the cheek samples, *Rothia.3* showed the greatest increase, while *Campylobacter* and *Lautropia* showed the greatest decrease, over time.

## CONCLUSIONS AND IMPLICATIONS

Our findings show that variation in the infant skin microbiome was driven largely by body site, with differences in bacterial alpha diversity emerging as early as the first hours of life. Common hospital practices that engender novel components of the early life environment may influence the initial development of the infant skin microbiome, as evidenced by associations between infant skin bacterial abundances and hospital bathing, as well as the administration of antibiotics to mothers and newborns. Our results also showed that the infant skin microbiome becomes more differentiated over time, with stronger differences in bacterial composition across body sites six weeks after birth compared to after delivery. The data suggest that during infancy, interactions with caregivers, as well as infant feeding and bathing practices, shape bacterial exposures. We also present evidence that the maternal skin microbiome may be impacted by hospital practices and shift during the postpartum period. By using an ecological and evolutionary lens to investigate relationships between hygiene and household factors and the skin microbiome, we highlight the potential for infants (and their microbes) to experience mismatches between ‘evolutionarily expected’ versus ‘evolutionarily novel’ microbial environments.

### Impact of hospital practices on the skin microbiome

In support of our first hypothesis, we detected variation in both bacterial alpha diversity and abundance profiles by body site at T1, suggesting differentiation of the infant skin microbiome even in the first hours of life. Infant axilla samples displayed the lowest bacterial alpha diversity, a finding in line with our group’s previous work on the skin microbiome in early life [42, 65] and adulthood [66]. Low bacterial alpha diversity in adults is thought to be driven by local ecological properties of axillary skin, including a density of sweat glands and hair, as well as behavioral factors like the use of deodorants and antiperspirants [67]. However, these factors are not relevant to infant skin, which makes our observation of variation in alpha diversity and abundance profiles across skin sites shortly after birth particularly intriguing. One explanation is that not all regions of infant skin receive the same exposure to maternal microbes during delivery. The axillae may be more ‘protected’ due to their anatomical position, compared to skin sites on the face, torso, or extremities that make direct contact with the birth canal. Further, most infants in the current study were swaddled when sample collection began at T1, such that their axilla and hands, but not cheeks, were covered by a blanket. This common hospital practice may provide a barrier to bacterial exposure and could explain why the cheek—a more ‘exposed’ body site—displayed the highest bacterial alpha diversity at T1. Future work is needed to better characterize potential mismatch scenarios, such as the use of clothing and blankets as barriers to initial exposure to the microbial ‘old friends’ present in the birth environment. We look forward to studies that evaluate the efficacy of practices like skin-to-skin contact to counteract the potential reduction in microbial exposures that occurs in evolutionarily novel birth environments.

Newborn skin is initially covered by the vernix caseosa (‘vernix’), which is composed of water, lipids and proteins, develops during the third trimester of pregnancy, and provides antibacterial properties and a pH of roughly 6–7 at birth [68–70]. Studies of vernix retention (rather than immediate removal) show that this protective layer can increase newborn skin hydration while decreasing its pH—two factors to which bacteria are sensitive [71, 72]. A relationship between the vernix and bacterial colonization has been proposed [73] but not directly tested. Evidence that vernix coverage varies by body site [68] suggests one intrinsic physiological mechanism for variation in skin bacterial alpha diversity across body sites following birth. Since both maternal and paternal biology can shape placental development [74], which in turn influences vernix formation [75], there may be unexplored evolutionary dynamics between parental biology, vernix coverage and the early life skin microbiome.

Hospital bathing was a significant predictor of overall bacterial abundance profiles on the infant cheek and hand at T1, further supporting our first hypothesis. Bathing newborns in the hospital during the first day of life is an evolutionarily novel practice that is in opposition to practices in many contemporary settings [76–78], yet was a routine practice at the hospital where participants were recruited. Washing infants may remove microbes from the skin [32, 79] alongside the vernix, and/or modify properties of the skin to which bacteria are sensitive, such as pH or moisture content [71, 72]. This suggests a scenario in which hospital hygiene practices contribute to the reduction of evolutionarily expected microbes from the birth and early life environment, a phenomenon in line with the Disappearing Microbiota Hypothesis [25]. More work is needed to identify how the disappearance of microbes from the birth environment may impair microbiome-mediate immune system development.

Hospital hygiene practices that affect infant skin bacterial communities, may allow environmentally sourced microbes to colonize the altered niches of newborn skin [80, 81]. This could explain our finding of a positive correlation between hospital bathing and the abundance of *Enterococcaceae* on the infant axilla, as well as the abundance of *Rhizobacteriaceae* (a bacterial family frequently associated with plants) on the infant hand at T1. These early colonization dynamics may have subsequent effects on bacterial community growth, composition and function—a phenomenon known as *priority effects* [82, 83]. While early bathing has value for infants who are born in settings where the risk of infection at birth is high [84], it is our hope that future studies will consider the development of the infant skin microbiome when evaluating the necessity of early bathing in settings where pathogen exposure is low. More work is needed to test how the exact timing or intensity (i.e. duration of bath, rigor of washing and product use) of the first bath impacts early microbial colonizers on the skin, and if this hygiene practice creates a mismatch scenario by limiting the colonization of particular microbes on the skin.

We found an association between maternal and infant antibiotic exposure and infant bacterial community composition at T1, and that maternal antibiotics were correlated to the abundances of *Staphylococcaceae* (negative) and *Rhizobiaceae* (positive) on multiple infant skin sites. An ecological perspective suggests that antibiotics impact the *regional species pool* of maternal microbes that are available to colonize the infant body during delivery and subsequent maternal-infant contact. This could explain the negative correlation between maternal antibiotics and the abundance of *Staphylococcaceae*, a dominant taxon on adult skin [85, 86]. Furthermore, newborn antibiotic exposure at birth was moderately correlated to the abundances of *Staphylococcaceae* and *Peptostreptococcales-Tissierellales* on the infant axilla at T2, suggesting a persistent impact of early-life antibiotics. It is also possible that antibiotic exposure in the hospital continued after discharge; if the dose concluded prior to the T2 study visit, then our survey (which asked about antibiotic use at the time of sample collection), would not have captured this potential recurring influence on infant skin.

The current study expands on our understanding of the impact of antibiotics on the infant gut microbiome [87] and to the best of our knowledge, is the first to document a relationship between antibiotics during delivery and the infant skin microbiome. Taken together, our findings related to both hospital bathing and antibiotic exposure are applicable to concerns that emerge from the Old Friends Hypothesis [23]—namely the potential for disrupted immune system development when infants have reduced exposure to the immune-modulating bacteria with which humans evolved. Future studies that apply this perspective can identify potential mismatches that arise due to the evolutionarily novel practices surrounding infant birth that occur in many industrialized contexts like the one in which the current study was conducted.

### Differential exposure to bacteria in the surrounding environment likely continues into infancy

Body site was an important predictor of variation in bacterial community composition as well as bacterial abundance profiles at T2. In support of our second hypothesis, the data suggest that the infant skin microbiome at 6 weeks of age is shaped by social and household environments. There was a trending relationship between receiving allocare and the microbiome of both the infant axilla (bacterial abundance profiles) and cheek (bacterial community composition) (*P*-value = 0.067 and 0.057, respectively). This is in line with previous work from our group which showed that the relationship between allocare and the infant skin microbiome varied by specific caregiving behaviors and infant body site [42]. While conducting this study during the COVID-19 pandemic precluded opportunities for behavioral observations of infant-caregiver interactions, physical contact during allocare could promote bacterial sharing with infants. Allocare is a hallmark of the human life history and child-rearing strategy [36–38], and may confer additional benefits to infants by diversifying the community of microbes that are available for dispersal to the infant body.

Anecdotal conversations with study families revealed that infants’ interactions with alloparents (particularly grandparents) were limited due to the COVID-19 pandemic. Travel restrictions and the unknown benefits of maternal vaccination for infant immunity at the time of the study, as well as parents’ ability to work from home and not rely on daycare or additional caregivers, were frequently mentioned as factors that restricted infants’ social environments. However, these behavioral changes may have increased opportunities for maternal-infant contact during a period in which bonding is crucial [88, 89], potentially facilitating direct breastfeeding (as opposed to feeding with expressed milk or formula). In post-pandemic studies, additional factors that underlie the availability of alloparents (e.g. living away from extended family; financial barriers to hiring caregivers) should be interrogated. Given the influence of sociocultural norms on allocare [90, 91], there is ample opportunity to investigate how larger societal forces create variation in infant-alloparent interactions. Since host genetics may influence the types of environmentally sourced bacteria that colonize the infant gut [92], it is possible that similar mechanisms modulate the development of the infant skin microbiome, such that bacterial sharing varies based on the biological relatedness of infants and caregivers. This has intriguing implications for potential mismatches that arise when infants acquire microbes during bouts of allocare from non-kin, such as nannies or daycare providers.

Having siblings in the household was significantly associated with bacterial community composition of the infant cheek, but not with other measures of the microbiome. This somewhat contradicts previous studies of the infant gut microbiome that consistently found a relationship to the presence of siblings in the household [44, 93–96]. One potentially important distinction of our study is that the COVID-19 pandemic limited siblings’ attendance, and associated bacterial exposures, at daycare or after-school activities. As such, siblings may have had a limited role as ‘vectors’ that introduced their family members, including infant siblings, to bacteria originating outside of the household.

Social environments may also indirectly impact the infant microbiome by shaping the microbiomes of mothers, including that of human milk [97]. Exclusive breastfeeding and pumping were strongly correlated to *Staphylococcaceae* abundance on the infant cheek (negatively and positively, respectively), a body site that frequently contacts milk during feeding. The opposing directions of these correlations suggest differences in infants’ bacterial exposures based on feeding mode, and are in line with previous findings of variation in the microbiome of pumped milk versus direct breast expression [98, 99]. Exclusive breastfeeding was also moderately-to-strongly negatively correlated to *Staphylococcaceae* abundance on the infant axilla and hand, and was also a significant predictor of infant cheek bacterial community composition. While *Staphylococcaceae* is a key member of the adult skin microbiome [85, 86], this family contains pathogenic species that directly contribute to skin conditions like atopic dermatitis in early life [100]. It may be that the protective effects of breastfeeding for infant health [101] extend to mitigating the risk of pathogenic *Staphylococcaceae* establishment on infant skin. In support of this idea, we found that *Staphylococcaceae* abundance on the cheek was only weakly negatively correlated to formula feeding, an evolutionarily novel practice that does not directly expose infants to the potentially protective microbes from maternal breast skin or human milk. There remains much to learn about connections between human milk feeding and the infant skin microbiome, including if decreased exposure to maternal microbes through formula and/or pumped milk feeding has health consequences, as put forth by the Disappearing Microbiota Hypothesis [25].

### Temporal changes in the skin microbiome differ across maternal and infant body sites

In support of our third hypothesis, we found that both the infant and maternal skin microbiome varied over time. Across all samples, there was a decrease in the prevalence of *Lactobacillus*, a taxon associated with vaginal delivery that may naturally be replaced by other taxa following childbirth. Though bacterial alpha diversity and richness increased to the greatest extent in the infant axilla, it was the body site with the lowest bacterial alpha diversity at both timepoints. In contrast, the infant cheek and hand displayed less variation in bacterial alpha diversity and richness over time, and harbored higher abundances of *Streptococcaceae* compared to the axilla at both timepoints. Because this taxon is a known member of the oral microbiome, the frequency with which infants’ hands touch their mouths and cheeks during ‘self-contact behaviors’ [102] may contribute to spreading *Streptococcaceae* to these body sites. Similarly, the prevalence of *Veillonella*, another taxon associated with the oral microbiome, increased considerably over time in the infant hand and cheek samples, but not the axilla samples. Infant samples clustered by body site (based on compositional abundance data) at both time points, though this trend was stronger at T2. At both timepoints, this grouping was driven by the axilla samples, further supporting the conclusion that the unique bacterial composition of the axilla microbiome emerges early in life [42, 65]. In addition to lower *Streptococcaceae* abundance, axilla samples also harbored lower abundances of *Propionibacteriaceae*—a bacterial family that contains taxa known to dominate facial skin [103].

Maternal samples showed a somewhat different pattern. All three body sites had higher bacterial alpha diversity and richness at T2 than at T1, though this change was more modest in the cheek and chest samples compared to the hand. Our finding of drastically higher maternal hand bacterial alpha diversity and richness at T2 suggests two, non-mutually exclusive explanations. First, it is possible that our data reflect a true increase in bacterial alpha diversity and richness over time as mothers acquire multiple types of bacteria on their hands, perhaps from interacting with people (including their infants) and surfaces in the household. It is also plausible that the T2 samples represent the ‘normal’ maternal skin microbiome, while the samples collected at T1 reflect changes to the skin microbiome that occur during hospitalization. Hospital hygiene practices such as handwashing and the use of antimicrobial sanitizers could impact the maternal hand microbiome to a greater degree than the chest or cheek (i.e. sanitizers are rarely applied to these body sites), though we were unable to observe these practices in the current study. In the likely event that hand hygiene was more stringent in the hospital than at home during the postpartum period, this would explain our finding that the bacterial alpha diversity and richness of the maternal hand microbiome was drastically lower at T1 compared to T2.

Relative to the infant samples, maternal skin samples showed a weaker pattern of clustering by body site at both timepoints. This result was unexpected, given the literature that highlights the adult skin microbiome as a collection of distinct bacterial communities scattered across various body sites [104–106]. However, our findings were generated using bacterial abundance data, rather than alpha diversity, richness, or other compositional metrics that have been deployed in other studies (some of which require larger sample sizes than what was available here). One interpretation of the current result of minimal variation in bacterial abundance is that changes in the maternal skin microbiome in the first 6 weeks of the postpartum period are driven more by dynamics related to the acquisition and/or loss of bacterial taxa rather than shifts in abundances. This idea is corroborated by our finding of significant differences in average bacterial prevalence over time (all maternal body sites combined as well as in the hand and chest samples). Of note, *Gemellaceae* prevalence increased in infant and maternal samples over time, and surpassed the prevalence threshold for inclusion in abundance analyses at T2, but not at T1. *Gemellaceae* has been associated with the human oral cavity [107] and detected in the human milk microbiome (unpublished data). It is possible that the prevalence and abundance patterns of *Gemellaceae* at T2 are related to contact between maternal bodies and the infant oral microbiome, potentially facilitated by breastfeeding.

Studies of the infant gut microbiome suggest a period of rapid bacterial acquisition in early life, often driven by direct bacterial transfer from maternal bodies [9]. If this phenomenon holds true for the skin, then we might expect to see strong temporal shifts in the presence or absence of most taxa detected on the infant skin. Instead, we found significant differences in average bacterial ASV prevalence only in the infant axilla samples, suggesting that bacterial dynamics on the skin may be governed by different ecological processes than those that operate in the gut [108]. Though empirical data are sparse, it has been suggested that bacterial communities on the skin are perpetually in a state of flux, perhaps due to the skin’s constant exposure to bacteria in the surrounding environment [102] and that a bacterial community sampled at one point in time is a product of multiple events of bacterial acquisition, loss and intra-community dynamics [79]. This could explain why we saw stronger signals related to shifting bacterial alpha diversity, richness and abundance, rather than the presence/absence of specific taxa, over time in the infant skin samples. Time-series data from frequent sample collection events will help disentangle longitudinal patterns of skin microbial diversity [108].

### Strengths, limitations and future directions

This study generated novel data on the bacterial communities of multiple skin sites, collected from mothers and newborns shortly after delivery and again at 6 weeks postpartum. The coupling of biological samples with questionnaire data allowed us to test for associations between the infant skin microbiome and common hospital practices, as well as factors related to the household and social environment during early life. To the best of our knowledge, this is the first study to explore associations between the infant skin microbiome and different early life hygiene practices (i.e. hospital bath; bath frequency and recency during infancy). This study also provided some of the first longitudinal skin swab samples collected from mother–infant dyads during the postpartum period. We stress the importance of appreciating human skin as a diverse microbial metacommunity [102] that can only be properly studied by collecting samples from the different niches found across body sites.

Chief among the limitations of the current study was sample size; replicating this study with a larger population would allow for more sophisticated analytical techniques and help confirm if the trends presented in this paper are indicative of strong statistical associations that may emerge only with larger sample sizes. While there were no significant differences in participant demographics across the two timepoints (Supplementary Table S1), it is possible that unmeasured variables differed between the two groups due to participant attrition. This could contribute to the differences in the microbiome between T1 and T2 that we report here. Future studies would benefit from including behavioral observations of infant-alloparent interactions to fully characterize the nature, frequency and duration of allocare needed for bacterial transmission. Due to the nature of conducting this study during the COVID-19 pandemic, we were limited in our ability to observe infant baths at the hospital or at home. Detailed information on infant bathing practices, from the timing of the first bath to the soap and rigor of scrubbing applied to infants’ skin, is needed to experimentally test for the influence of these hygiene practices on the infant skin microbiome. Long-read bacterial sequencing would provide enhanced taxonomic resolution of the data, allowing researchers to better infer patterns of bacterial sharing between infants, caregivers and the surrounding environment. Intentional participant recruitment to include families of varied structures and lifestyles will also be beneficial, as our results suggest opportunities for engaging more deeply with the cultural and behavioral dimensions of infant caregiving. We look forward to future studies that use similar analytical methods (i.e. clr-transformed ASV abundances) and study design (skin swab samples from the cheek, hand and axilla of full-term infants born vaginally), such that our results, especially those related to bacterial diversity and abundances, can be compared to findings in other cohorts.

## CONCLUSION

The current study provides new evidence that common hospital practices such as newborn bathing and exposure to antibiotics may influence the initial development of the infant skin microbiome. Our data also suggest that interactions with alloparents, as well as practices related to infant hygiene and feeding, are factors worth considering as drivers of skin microbiome development in early life. These results add to the current body of literature exploring the early life skin [42] and gut [43, 44, 109] microbiomes in the context of household and social environments. Given the considerable variation in birthing and early life environments that exist across human geographic and sociocultural settings, the results of this study highlight the need for future research that interrogates how these differences connect the microbiome to early life health inequities [110–112]. Integrating measures of immune system activity and infant growth will also be beneficial. Since the acquisition of microbes from the early life environment directly shapes immune system expansion [3, 113], changes and/or reductions to the microbial milieu of infancy, including those reported here, are likely linked to the increasing rates of immune system dysregulation in settings with stringent hygiene practices and reduced rates of breastfeeding. Given the importance of *timing* for the assembly of microbial communities in open niches [82, 83], and our observation of differences in infant skin bacterial communities in relation to bathing and antibiotic use in the first hours of life, we suspect that environmental influences on the co-development of the infant microbiome and immune system begin during and immediately following delivery. Moving forward, studies of the skin microbiome should measure the production of short-chain fatty acids (SCFAs), microbial metabolites that regulate immune activity [114]. This will help confirm if the taxonomic variation in skin bacterial communities that we report here impacts infant immunity via corresponding functional differences in the production of SCFAs. We look forward to future studies that foreground evolutionary considerations of the microbiome within the context of human life history and social child-rearing strategies, as well as identify instances where infant hygiene and caregiving practices may produce mismatches that impact the co-developing microbiome and immune system during early life.

## Supplementary Material

eoae023_suppl_Supplementary_Appendix

eoae023_suppl_Supplementary_Table

## References

[1] Ahmed T, Auble D, Berkley JA et al An evolving perspective about the origins of childhood undernutrition and nutritional interventions that includes the gut microbiome. Ann N Y Acad Sci 2014;1332:22–38. 25118072 10.1111/nyas.12487 PMC4514967

[2] Dowd JB, Renson A. ‘Under the Skin’ and into the gut: social epidemiology of the microbiome. Curr Epidemiol Rep 2018;5:432–41. 30596004 10.1007/s40471-018-0167-7 PMC6290701

[3] Gensollen T, Iyer SS, Kasper DL et al How colonization by microbiota in early life shapes the immune system. Science 2016;352:539–44. 27126036 10.1126/science.aad9378 PMC5050524

[4] Yang I, Corwin EJ, Brennan PA et al The infant microbiome: implications for infant health and neurocognitive development. Nurs Res 2016;65:76–88. 26657483 10.1097/NNR.0000000000000133 PMC4681407

[5] de Goffau MC, Lager S, Sovio U et al Human placenta has no microbiome but can contain potential pathogens. Nature 2019;572:329–34. 31367035 10.1038/s41586-019-1451-5 PMC6697540

[6] Kuperman A, Zimmerman A, Hamadia S et al Deep microbial analysis of multiple placentas shows no evidence for a placental microbiome. BJOG 2020;127:159–69. 31376240 10.1111/1471-0528.15896

[7] Perez-Muñoz ME, Arrieta M-C, Ramer-Tait AE et al A critical assessment of the ‘sterile womb’ and ‘in utero colonization’ hypotheses: implications for research on the pioneer infant microbiome. Microbiome 2017;5:48. 28454555 10.1186/s40168-017-0268-4 PMC5410102

[8] Dominguez-Bello MG, Costello EK, Contreras M et al Delivery mode shapes the acquisition and structure of the initial microbiota across multiple body habitats in newborns. Proc Natl Acad Sci USA 2010;107:11971–5. 20566857 10.1073/pnas.1002601107 PMC2900693

[9] Ferretti P, Pasolli E, Tett A et al Mother-to-infant microbial transmission from different body sites shapes the developing infant gut microbiome. Cell Host Microbe 2018;24:133–45.e5. 30001516 10.1016/j.chom.2018.06.005 PMC6716579

[10] Korpela K, Costea P, Coelho LP et al Selective maternal seeding and environment shape the human gut microbiome. Genome Res 2018;28:561–8. 29496731 10.1101/gr.233940.117 PMC5880245

[11] Bokulich NA, Chung J, Battaglia T et al Antibiotics, birth mode, and diet shape microbiome maturation during early life. Sci Transl Med 2016;8:343ra–82. 10.1126/scitranslmed.aad7121 PMC5308924 27306664

[12] Azad M, Konya T, Persaud R et al; the CHILD Study Investigators. Impact of maternal intrapartum antibiotics, method of birth and breastfeeding on gut microbiota during the first year of life: a prospective cohort study. BJOG 2016;123:983–93. 26412384 10.1111/1471-0528.13601

[13] Ivanov II, Atarashi K, Manel N et al Induction of intestinal Th17 cells by segmented filamentous bacteria. Cell 2009;139:485–98. 19836068 10.1016/j.cell.2009.09.033 PMC2796826

[14] Cahenzli J, Köller Y, Wyss M et al Intestinal microbial diversity during early-life colonization shapes long-term IgE levels. Cell Host Microbe 2013;14:559–70. 24237701 10.1016/j.chom.2013.10.004 PMC4049278

[15] Naik S, Bouladoux N, Wilhelm C et al Compartmentalized control of skin immunity by resident commensals. Science 2012;337:1115–9. 22837383 10.1126/science.1225152 PMC3513834

[16] Wanke I, Steffen H, Christ C et al Skin commensals amplify the innate immune response to pathogens by activation of distinct signaling pathways. J Invest Dermatol 2011;131:382–90. 21048787 10.1038/jid.2010.328

[17] Mills JG, Selway CA, Thomas T et al Schoolyard biodiversity determines short-term recovery of disturbed skin microbiota in children. Microb Ecol 2022;86:658–69. DOI: https://doi.org/10.1007/s00248-022-02052-2 35689685 PMC9188306

[18] Roslund MI, Puhakka R, Grönroos M et al; ADELE research group. Biodiversity intervention enhances immune regulation and health-associated commensal microbiota among daycare children. Sci Adv 2020;6:eaba2578. 33055153 10.1126/sciadv.aba2578 PMC7556828

[19] Mulder IE, Schmidt B, Stokes CR et al Environmentally-acquired bacteria influence microbial diversity and natural innate immune responses at gut surfaces. BMC Biol 2009;7:79. 19930542 10.1186/1741-7007-7-79 PMC2785767

[20] Scharschmidt TC, Vasquez KS, Truong H-A et al A wave of regulatory T cells into neonatal skin mediates tolerance to commensal microbes. Immunity 2015;43:1011–21. 26588783 10.1016/j.immuni.2015.10.016 PMC4654993

[21] Kuzawa CW, Quinn EA. Developmental origins of adult function and health: evolutionary hypotheses. Annu Rev Anthropol 2009;38:131–47.

[22] Strachan DP. Hay fever, hygiene, and household size. BMJ 1989;299:1259–60. 2513902 10.1136/bmj.299.6710.1259 PMC1838109

[23] Rook GAW, Lowry CA, Raison CL. Microbial ‘Old Friends’, immunoregulation and stress resilience. Evol. Med. Public Health 2013;2013:46–64. 24481186 10.1093/emph/eot004 PMC3868387

[24] Rook GAW, Raison CL, Lowry CA. Microbial ‘old friends’, immunoregulation and socioeconomic status. Clin Exp Immunol 2014;177:1–12. 24401109 10.1111/cei.12269 PMC4089149

[25] Blaser MJ, Falkow S. What are the consequences of the disappearing human microbiota? Nat Rev Microbiol 2009;7:887–94. 19898491 10.1038/nrmicro2245 PMC9354563

[26] Hanski I, von Hertzen L, Fyhrquist N et al Environmental biodiversity, human microbiota, and allergy are interrelated. Proc Natl Acad Sci U S A 2012;109:8334–9. 22566627 10.1073/pnas.1205624109 PMC3361383

[27] Ownby DR, Johnson CC, Peterson EL. Exposure to dogs and cats in the first year of life and risk of allergic sensitization at 6 to 7 years of age. JAMA 2002;288:963–72. 12190366 10.1001/jama.288.8.963

[28] Riedler J, Braun-Fahrländer C, Eder W et al; ALEX Study Team. Exposure to farming in early life and development of asthma and allergy: a cross-sectional survey. Lancet 2001;358:1129–33. 11597666 10.1016/S0140-6736(01)06252-3

[29] Ruokolainen L, von Hertzen L, Fyhrquist N et al Green areas around homes reduce atopic sensitization in children. Allergy 2015;70:195–202. 25388016 10.1111/all.12545 PMC4303942

[30] Manus MB. Evolutionary mismatch. Evol. Med. Public Health 2018;2018:190–1. 30159142 10.1093/emph/eoy023 PMC6109377

[31] Corbett S, Courtiol A, Lummaa V et al The transition to modernity and chronic disease: mismatch and natural selection. Nat Rev Genet 2018;19:419–30. 29743650 10.1038/s41576-018-0012-3

[32] Yu JJ, Manus MB, Mueller O et al Antibacterial soap use impacts skin microbial communities in rural Madagascar. PLoS One 2018;13:e0199899. 30125279 10.1371/journal.pone.0199899 PMC6101359

[33] Donovan BM, Abreo A, Ding T et al Dose, timing, and type of infant antibiotic use and the risk of childhood asthma. Clin Infect Dis 2020;70:1658–65. 31149702 10.1093/cid/ciz448 PMC7145998

[34] Bailey LC, Forrest CB, Zhang P et al Association of antibiotics in infancy with early childhood obesity. JAMA Pediatr 2014;168:1063–9. 25265089 10.1001/jamapediatrics.2014.1539

[35] Andrikopoulou M, Huang Y, Duffy CR et al Antibiotic use without indication during delivery hospitalizations in the United States. Obstet Gynecol 2019;134:718–25. 31503161 10.1097/AOG.0000000000003485 PMC6768706

[36] Hrdy SB. Mothers and Others: The Evolutionary Origins of Mutual Understanding. Cambridge, MA, USA: Harvard University Press, 2009.

[37] Hrdy SB. Comes the child before man: how cooperative breeding and prolonged postweaning dependence shaped human potential. Hunt-Gatherer Childhoods 2017. DOI: https://doi.org/10.4324/9780203789445-4

[38] Kramer KL. Cooperative breeding and its significance to the demographic success of humans. Annu Rev Anthropol 2010;39:417–36.

[39] Lucas FS, Moureau B, Jourdie V et al Brood size modifications affect plumage bacterial assemblages of European starlings. Mol Ecol 2005;14:639–46. 15660952 10.1111/j.1365-294X.2005.02436.x

[40] Yarlagadda K, Razik I, Malhi RS et al Social convergence of gut microbiomes in vampire bats. Biol Lett 2021;17:20210389. 34727703 10.1098/rsbl.2021.0389 PMC8563296

[41] Ross AA, Doxey AC, Neufeld JD. The skin microbiome of cohabiting couples. MSystems 2017;2:e00043–17. 28761935 10.1128/mSystems.00043-17 PMC5527301

[42] Manus MB, Sardaro MLS, Dada O et al Interactions with alloparents are associated with the diversity of infant skin and fecal bacterial communities in Chicago, United States. Am J Hum Biol 2023;n/a:e23972. 10.1002/ajhb.23972 PMC11667966 37632331

[43] Wiley KS, Gregg AM, Fox MM et al Contact with caregivers is associated with composition of the infant gastrointestinal microbiome in the first 6 months of life. Am J Biol Anthropol 2024;183:e24858. 37804008 10.1002/ajpa.24858 PMC10922139

[44] Manus MB, Watson E, Kuthyar S et al Prenatal household size and composition are associated with infant fecal bacterial diversity in Cebu, Philippines. Am J Biol Anthropol 2023;181:45–58. 36847111 10.1002/ajpa.24720

[45] Domingues CPF, Rebelo JS, Dionisio F et al The social distancing imposed to contain COVID-19 can affect our microbiome: a double-edged sword in human health. MSphere 2020;5:e00716–20. DOI: https://doi.org/10.1128/mSphere.00716-20 32938697 PMC7494832

[46] Finlay BB, Amato KR, Azad M et al The hygiene hypothesis, the COVID pandemic, and consequences for the human microbiome. Proc Natl Acad Sci USA 2021;118:e2010217118. 33472859 10.1073/pnas.2010217118 PMC8017729

[47] McCall L-I, Callewaert C, Zhu Q et al Home chemical and microbial transitions across urbanization. Nat Microbiol 2020;5:108–15. 31686026 10.1038/s41564-019-0593-4 PMC7895447

[48] Combellick JL, Shin H, Shin D et al Differences in the fecal microbiota of neonates born at home or in the hospital. Sci Rep 2018;8:15660. 30353125 10.1038/s41598-018-33995-7 PMC6199260

[49] Janulis P, Phillips G, Melville J et al Network canvas: an open-source tool for capturing social and contact network data. Int J Epidemiol 2023;52:1286–91. 36944105 10.1093/ije/dyad036 PMC10396415

[50] Dhudasia MB, Flannery DD, Pfeifer MR et al Updated guidance: prevention and management of perinatal group B streptococcus infection. Neoreviews 2021;22:e177–88. 33649090 10.1542/neo.22-3-e177

[51] Thompson LR, Sanders JG, McDonald D et al; Earth Microbiome Project Consortium. A communal catalogue reveals Earth’s multiscale microbial diversity. Nature 2017;551:457–63. 29088705 10.1038/nature24621 PMC6192678

[52] Mallott EK, Amato KR. The microbial reproductive ecology of white-faced capuchins (*Cebus capucinus*). Am J Primatol 2018;80:e22896. 29984842 10.1002/ajp.22896

[53] Walters W, Hyde ER, Berg-Lyons D et al Improved bacterial 16S rRNA Gene (V4 and V4-5) and fungal internal transcribed spacer marker gene primers for microbial community surveys. MSystems 2016;1:e00009–15. 10.1128/mSystems.00009-15 PMC5069754 27822518

[54] Bolyen E, Rideout JR, Dillon MR et al Reproducible, interactive, scalable and extensible microbiome data science using QIIME 2. Nat Biotechnol 2019;37:852–7. 31341288 10.1038/s41587-019-0209-9 PMC7015180

[55] McMurdie PJ, Holmes S. Waste not, want not: why rarefying microbiome data is inadmissible. PLoS Comput Biol 2014;10:e1003531. 24699258 10.1371/journal.pcbi.1003531 PMC3974642

[56] Willis AD. Rarefaction, alpha diversity, and statistics. Front Microbiol 2019;10:2407. 31708888 10.3389/fmicb.2019.02407 PMC6819366

[57] McMurdie PJ, Holmes S. phyloseq: an R package for reproducible interactive analysis and graphics of microbiome census data. PLoS One 2013;8:e61217. 23630581 10.1371/journal.pone.0061217 PMC3632530

[58] R Core Team. R: a language and environment for statistical computing. R Foundation for Statistical Computing, Vienna, Austria. 2023. https://www.R-project.org/

[59] Wickham H. Ggplot2: Elegant Graphics for Data Analysis. Springer-Verlag New York. 2016.

[60] Ahlmann-Eltze C, Patil I.. ggsignif: R package for displaying significance brackets for ‘ggplot2’. 2021, DOI: https://doi.org/10.31234/osf.io/7awm6

[61] Anderson MJA. new method for non-parametric multivariate analysis of variance. Austral Ecol 2001;26:32–46.

[62] Oksanen J, Simpson G, Blanchet F et al vegan: community ecology package. R package version 2.6.4. 2022.

[63] Barnett DJ, Arts IC, Penders J. microViz: an R package for microbiome data visualization and statistics. J Open Source Softw 2021;6:3201.

[64] Conway JR, Lex A, Gehlenborg N. UpSetR: an R package for the visualization of intersecting sets and their properties. Bioinformatics 2017;33:2938–40. 28645171 10.1093/bioinformatics/btx364 PMC5870712

[65] Manus MB, Kuthyar S, Perroni-Marañón AG et al Infant skin bacterial communities vary by skin site and infant age across populations in Mexico and the United States. MSystems 2020;5:1. DOI: https://doi.org/10.1128/msystems.00834-20 PMC7646528 33144313

[66] Manus MB, Yu JJ, Park LP et al Environmental influences on the skin microbiome of humans and cattle in rural Madagascar. Evol. Med. Public Health 2017;2017:144–53. 29147568 10.1093/emph/eox013 PMC5631097

[67] Urban J, Fergus DJ, Savage AM et al The effect of habitual and experimental antiperspirant and deodorant product use on the armpit microbiome. PeerJ 2016;4:e1605. 26855863 10.7717/peerj.1605 PMC4741080

[68] Visscher MO, Narendran V, Pickens WL et al Vernix caseosa in neonatal adaptation. J Perinatol 2005;25:440–6. 15830002 10.1038/sj.jp.7211305

[69] Kutlubay Z, Tanakol A, Engýn B et al Newborn skin: common skin problems. Maedica 2017;12:42–7. 28878836 PMC5574071

[70] Visscher MO, Adam R, Brink S et al Newborn infant skin: physiology, development, and care. Clin Dermatol 2015;33:271–80. 25889127 10.1016/j.clindermatol.2014.12.003

[71] Zhalnina K, Dias R, de Quadros PD et al Soil pH determines microbial diversity and composition in the park grass experiment. Microb Ecol 2015;69:395–406. 25395291 10.1007/s00248-014-0530-2

[72] Akaza N, Takasaki K, Matsudaira T et al Relationship between skin fungal and bacterial microbiomes and skin pH. Int J Cosmet Sci 2023;45:362–72. 36752033 10.1111/ics.12842

[73] Hoath SB, Pickens WL, Visscher MO. The biology of vernix caseosa. Int J Cosmet Sci 2006;28:319–33. 18489296 10.1111/j.1467-2494.2006.00338.x

[74] Bhadsavle SS, Golding MC. Paternal epigenetic influences on placental health and their impacts on offspring development and disease. Front Genet 2022;13:1068408. 36468017 10.3389/fgene.2022.1068408 PMC9716072

[75] Visscher M, Narendran V. Vernix caseosa: formation and functions. Newborn Infant Nurs Rev 2014;14:142–6.

[76] Mrayan L, Abujilban S, Abuidhail J et al Traditional neonatal care practices in Jordan: a qualitative study. Nurs Health Sci 2018;20:486–93. 29947465 10.1111/nhs.12540

[77] Kayom VO, Kakuru A, Kiguli S. Newborn care practices among mother-infant dyads in urban Uganda. Int J Pediatr 2015;2015:1–8. 10.1155/2015/815938 PMC4680047 26713096

[78] Priyadarshi M, Balachander B, Gupta S et al Timing of first bath in term healthy newborns: a systematic review. J Glob Health 2022;12:12004. 35972992 10.7189/jogh.12.12004 PMC9380966

[79] Vandegrift R, Fahimipour AK, Muscarella M et al Moving microbes: the dynamics of transient microbial residence on human skin. *BioRXiv* 2019:586008.

[80] Brooks B, Olm MR, Firek BA et al Strain-resolved analysis of hospital rooms and infants reveals overlap between the human and room microbiome. Nat Commun 2017;8:1814. 29180750 10.1038/s41467-017-02018-w PMC5703836

[81] Rampelotto PH, Sereia AFR, de Oliveira LFV et al Exploring the hospital microbiome by high-resolution 16S rRNA profiling. Int J Mol Sci 2019;20:3099. 31242612 10.3390/ijms20123099 PMC6696720

[82] Fukami T. Historical contingency in community assembly: integrating niches, species pools, and priority effects. Annu Rev Ecol Evol Syst 2015;46:1–23.

[83] Debray R, Herbert RA, Jaffe AL et al Priority effects in microbiome assembly. Nat Rev Microbiol 2021;20:109–21. 34453137 10.1038/s41579-021-00604-w

[84] Medves JM, O’Brien B. Does bathing newborns remove potentially harmful pathogens from the skin? Birth 2001;28:161–5. 11552963 10.1046/j.1523-536x.2001.00161.x

[85] Severn MM, Williams MR, Shahbandi A et al The ubiquitous human skin commensal staphylococcus hominis protects against opportunistic pathogens. mBio 2022;13:e0093022. 35608301 10.1128/mbio.00930-22 PMC9239047

[86] Zheng Y, Hunt RL, Villaruz AE et al Commensal *Staphylococcus epidermidis* contributes to skin barrier homeostasis by generating protective ceramides. Cell Host Microbe 2022;30:301–13.e9. 35123653 10.1016/j.chom.2022.01.004 PMC8917079

[87] Dunn AB, Jordan S, Baker BJ et al The maternal infant microbiome: considerations for labor and birth. MCN Am J Matern Child Nurs 2017;42:318–25. 28825919 10.1097/NMC.0000000000000373 PMC5648605

[88] Liu J, Leung P, Yang A. Breastfeeding and active bonding protects against children’s internalizing behavior problems. Nutrients 2014;6:76–89. 10.3390/nu6010076 PMC3916850 24368674

[89] Nagasawa M, Okabe S, Mogi K et al Oxytocin and mutual communication in mother-infant bonding. Front Hum Neurosci 2012;6:31. DOI: https://doi.org/10.3389/fnhum.2012.00031 22375116 PMC3289392

[90] Meehan CL. The effects of residential locality on parental and alloparental investment among the Aka foragers of the central African Republic. Hum Nat 2005;16:58–80. 26189516 10.1007/s12110-005-1007-2

[91] Rosenbaum S, Kuzawa CW, McDade TW et al Fathers’ care in context: ‘facultative’, flexible fathers respond to work demands and child age, but not to alloparental help, in Cebu, Philippines. Evol Hum Behav 2021;42:534–46.

[92] Tavalire HF, Christie DM, Leve LD et al Shared environment and genetics shape the gut microbiome after infant adoption. mBio 2021;12:e00548–21. 33785620 10.1128/mBio.00548-21 PMC8092250

[93] Azad MB, Konya T, Maughan H et al Infant gut microbiota and the hygiene hypothesis of allergic disease: impact of household pets and siblings on microbiota composition and diversity. Allergy Asthma Clin Immunol 2013;9:15. 23607879 10.1186/1710-1492-9-15 PMC3655107

[94] Laursen MF, Zachariassen G, Bahl MI et al Having older siblings is associated with gut microbiota development during early childhood. BMC Microbiol 2015;15:154. 26231752 10.1186/s12866-015-0477-6 PMC4522135

[95] Laursen MF, Laursen RP, Larnkjær A et al Faecalibacterium gut colonization is accelerated by presence of older siblings.. MSphere 2017;2:e00448–17. 29202044 10.1128/mSphere.00448-17 PMC5705805

[96] Hasegawa K, Linnemann RW, Mansbach JM et al Household siblings and nasal and fecal microbiota in infants. Pediatr Int 2017;59:473–81. 27638139 10.1111/ped.13168 PMC5354996

[97] Meehan CL, Lackey KA, Hagen EH et al Social networks, cooperative breeding, and the human milk microbiome. Am J Hum Biol 2018;30:e23131. 29700885 10.1002/ajhb.23131

[98] Moossavi S, Sepehri S, Robertson B et al Composition and variation of the human milk microbiota are influenced by maternal and early-life factors. Cell Host Microbe 2019;25:324–35.e4. 30763539 10.1016/j.chom.2019.01.011

[99] Fehr K, Moossavi S, Sbihi H et al Breastmilk feeding practices are associated with the co-occurrence of bacteria in mothers’ milk and the infant gut: the CHILD cohort study. Cell Host Microbe 2020;28:285–97.e4. 32652062 10.1016/j.chom.2020.06.009

[100] Huang K, Li F, Liu Y et al Multi-omics analyses reveal interactions between the skin microbiota and skin metabolites in atopic dermatitis. Front Microbiol 2024;15:1349674. DOI: https://doi.org/10.3389/fmicb.2024.1349674 38559353 PMC10978668

[101] Manus MB, Goguen SK, Azad MB. The protective associations of breastfeeding with infant overweight and asthma are not dependent on maternal FUT2 secretor status. Front Nutr 2023;10:1203552. 37964924 10.3389/fnut.2023.1203552 PMC10642293

[102] Manus MB. Ecological processes and human behavior provide a framework for studying the skin microbial metacommunity. Microb Ecol 2022;84:689–702. 34636925 10.1007/s00248-021-01884-8

[103] Bek-Thomsen M, Lomholt HB, Kilian M. Acne is not associated with yet-uncultured bacteria. J Clin Microbiol 2008;46:3355–60. 18716234 10.1128/JCM.00799-08 PMC2566126

[104] Grice EA, Kong HH, Conlan S et al; NISC Comparative Sequencing Program. Topographical and temporal diversity of the human skin microbiome. Science 2009;324:1190–2. 19478181 10.1126/science.1171700 PMC2805064

[105] Blaser MJ, Dominguez-Bello MG, Contreras M et al Distinct cutaneous bacterial assemblages in a sampling of South American Amerindians and US residents. ISME J 2013;7:85–95. 22895161 10.1038/ismej.2012.81 PMC3526177

[106] Perez GIP, Gao Z, Jourdain R et al Body site Is a more determinant factor than human population diversity in the healthy skin microbiome. PLoS One 2016;11:e0151990. 27088867 10.1371/journal.pone.0151990 PMC4835103

[107] Torres-Morales J, Mark Welch JL, Dewhirst FE et al Site-specialization of human oral Gemella species. J Oral Microbiol 2023;15:2225261. 37361319 10.1080/20002297.2023.2225261 PMC10288933

[108] Bogaert D, van Beveren GJ, de Koff EM et al Mother-to-infant microbiota transmission and infant microbiota development across multiple body sites. Cell Host Microbe 2023;31:447–60.e6. 36893737 10.1016/j.chom.2023.01.018

[109] Lane AA, McGuire MK, McGuire MA et al Household composition and the infant fecal microbiome: the INSPIRE study. Am J Phys Anthropol 2019;169:526–39. 31012086 10.1002/ajpa.23843

[110] Ishaq SL, Parada FJ, Wolf PG et al Introducing the microbes and social equity working group: considering the microbial components of social, environmental, and health justice. MSystems 2021;6:e0047121. 34313460 10.1128/mSystems.00471-21 PMC8407420

[111] Ishaq SL, Rapp M, Byerly R et al Framing the discussion of microorganisms as a facet of social equity in human health. PLoS Biol 2019;17:e3000536. 31770370 10.1371/journal.pbio.3000536 PMC6879114

[112] Amato KR, Arrieta M-C, Azad MB et al The human gut microbiome and health inequities. Proc Natl Acad Sci USA 2021;118:e2017947118. 34161260 10.1073/pnas.2017947118 PMC8237592

[113] Wiertsema SP, van Bergenhenegouwen J, Garssen J et al The interplay between the gut microbiome and the immune system in the context of infectious diseases throughout life and the role of nutrition in optimizing treatment strategies. Nutrients 2021;13:886. 33803407 10.3390/nu13030886 PMC8001875

[114] Traisaeng S, Herr DR, Kao H-J et al A Derivative of butyric acid, the fermentation metabolite of *Staphylococcus epidermidis*, inhibits the growth of a *Staphylococcus aureus* strain isolated from atopic dermatitis patients. Toxins 2019;11:311. 31159213 10.3390/toxins11060311 PMC6628397

