# Correction to: Birth and household exposures are associated with changes to skin bacterial communities during infancy

**DOI:** 10.1093/emph/eoaf024

**Published:** 2025-10-08

This is a correction to: Melissa B Manus, Maria Luisa Savo Sardaro, Omolola Dada, Maya Davis, Melissa R Romoff, Stephanie G Torello, Esther Ubadigbo, Rebecca C Wu, Maria Gloria Dominguez-Bello, Melissa K Melby, Emily S Miller, Katherine R Amato, Birth and household exposures are associated with changes to skin bacterial communities during infancy, *Evolution, Medicine, and Public Health*, Volume 13, Issue 1, 2025, Pages 49–76, https://doi.org/10.1093/emph/eoae023

Changes have been made to the figures within the originally-published manuscript to improve appearance and readability. Emendations have been made in Tables 4 and 7. altering stated values to read: ``<0.05''.

