# Supplementary material for: Birth and household exposures are associated with changes to skin bacterial communities during infancy
Source: Evol Med Public Health. 2024 Sep 17;13(1):49–76. doi: 10.1093/emph/eoae023 (PMC11966193; doi:10.1093/emph/eoae023)
Supplement: eoae023_suppl_Supplementary_Appendix [file eoae023_suppl_supplementary_appendix.docx]

**Appendix 1.** Modified laboratory protocol for bacterial DNA extraction from skin swab samples using the Qiagen DNeasy PowerSoil Pro kit.

1. Spin the PowerBead Pro Tube briefly to ensure that the beads have settled at the bottom. Add the skin swab head and trim the stem with sterilized scissors. Add 800 µL of Solution CD1. Vortex briefly to mix and heat at 70˚ C for 15 minutes.

2. Secure the PowerBead Pro Tube horizontally on a Vortex adapter and vortex at maximum speed for 15 minutes.

3. Centrifuge the PowerBead Pro Tube at 13,000 x g for 1 minute.

4. Transfer the supernatant to a clean 2 mL Microcentrifuge Tube.

5. Add 200 µL of Solution CD2 and vortex for 10 seconds.

6. Put tubes into 4˚ C fridge for 10 minutes.

7. Centrifuge at 13,000 x g for 1 minute. Avoiding the pellet, transfer up to 700 µL of supernatant to a clean 2 mL Microcentrifuge Tube.

8. Add 600 µL of Solution CD3 and vortex for 5 seconds.

9. Load 650 µL of the lysate onto an MB Spin Column and centrifuge at 13,000 x g for 30 seconds.

10. Discard the flow-through and repeat step 9 to ensure that all of the lysate has passed through the MB Spin Column.

11. Place the MB Spin Column into a clean 2 mL Collection Tube.

12. Add 500 µL of Solution EA to the MB Spin Column. Centrifuge at 13,000 x g for 1 minute.

13. Discard the flow-through and place the MB Spin Column back into the same 2 mL Collection Tube.

14. Add 500 µL of Solution C5 to the MB Spin Column. Centrifuge at 13,000 x g for 1 minute.

15. Discard the flow-through and place the MB Spin Column back into a new 2 mL Collection Tube.

16. Centrifuge at 13,000 x g for 2 minutes. Place the MB Spin Column into a new 1.6 mL Elution Tube.

17. Add 80 µL of Solution C6 to the center of the white filter membrane.

18. Centrifuge at 13,000 x g for 2 minutes. Discard the MB Spin Column. Store the DNA in the fridge or freezer.
