# Supplementary material for: Birth and household exposures are associated with changes to skin bacterial communities during infancy
Source: Evol Med Public Health. 2024 Sep 17;13(1):49–76. doi: 10.1093/emph/eoae023 (PMC11966193; doi:10.1093/emph/eoae023)
Supplement: eoae023_suppl_Supplementary_Table [file eoae023_suppl_supplementary_table.docx]

**Table S1.** Student's t-test revealed no statistically significant differences in participant demographics between T1 and T2.

| *Variable* | *p-value* |
| --- | --- |
| Infant sex | 0.43 |
| Maternal age at delivery years) | 0.35 |
| Maternal antibiotics during labor | 0.41 |
| Neonatal antibiotics | 0.36 |
| Hospital bath prior to sample collection | 0.36 |
| Current exclusive breastfeeding | 0.32 |
